# Artificial intelligence for early prediction of gestational diabetes mellitus and preeclampsia: a systematic review of machine learning models and clinical decision support systems

**DOI:** 10.3389/frai.2026.1890320

**Published:** 2026-07-30

**Authors:** Subhashree Barada, Ramani Selvanambi

**Affiliations:** School of Computer Science and Engineering, Vellore Institute of Technology, Vellore, Tamil Nadu, India

**Keywords:** artificial intelligence, clinical decision support systems, deep learning, gestational diabetes mellitus, machine learning, multi-modal learning, preeclampsia

## Abstract

Gestational diabetes mellitus (GDM) and preeclampsia are among the most significant pregnancy complications, affecting approximately 5–15% and 2–8% of pregnancies worldwide, respectively. These disorders share overlapping metabolic, vascular, inflammatory, and placental mechanisms, highlighting the need for integrated approaches to early prediction and risk assessment. However, existing artificial intelligence (AI)-based prediction models generally address GDM and preeclampsia independently and are often limited by inadequate multimodal data integration, insufficient external validation, and limited model interpretability. This systematic review synthesizes recent advances (2020–2026) in AI-based prediction of GDM and preeclampsia, with emphasis on predictive methodologies, data modalities, validation strategies, and potential clinical applications. The review was conducted in accordance with the PRISMA 2020 guidelines, and 120 studies employing machine learning (ML), deep learning (DL), and hybrid AI approaches using clinical, biochemical, electronic health record (EHR), and multimodal data were included. Across the reviewed studies, AI-based models demonstrated promising predictive performance, with reported area under the receiver operating characteristic curve (AUC) values ranging from 0.70 to 0.95. Ensemble and deep learning approaches generally outperformed conventional statistical methods, particularly when multimodal data were integrated. Frequently identified predictive variables included maternal clinical characteristics, metabolic biomarkers, inflammatory biomarkers, and angiogenic markers such as soluble fms-like tyrosine kinase-1 (sFlt-1) and placental growth factor (PlGF). Nevertheless, important methodological challenges remain, including limited external validation, substantial data heterogeneity, insufficient model interpretability, inconsistent reporting practices, and limited integration into routine clinical workflows. Furthermore, most existing AI models predict GDM or preeclampsia independently despite their shared pathophysiological mechanisms, highlighting an important gap in current prediction research. This review provides a comprehensive synthesis of epidemiological, clinical, mechanistic, and AI-based evidence and proposes an evidence-informed conceptual framework that integrates multimodal data, mechanism-aware modeling, explainable AI, standardized validation, and clinical decision-support considerations. Rather than representing a validated predictive system, the proposed framework provides a conceptual foundation to guide future AI model development, prospective validation, and clinical evaluation. Overall, the findings highlight key opportunities and remaining challenges for developing robust, interpretable, and generalizable AI-based prediction models to support future precision maternal healthcare and improve maternal and neonatal outcomes.

## 1 Introduction

Pregnancy is a complex physiological process characterized by remarkable changes in metabolism, hormones, and cardiovascular processes that promote fetal growth and its development. Regardless of these adaptive changes, a significant percentage of pregnancies are affected by complications that negatively impact the maternal and neonatal outcomes. Gestational diabetes mellitus (GDM) and preeclampsia are the two most prevalent and medically important complications, leading to increased maternal morbidity, poor perinatal outcomes, and long-term cardiometabolic disease risk (Barrell, 2025; Nguyen, 2024).

Gestational diabetes mellitus refers to glucose intolerance initially identified during pregnancy, which occurs when pancreatic beta cells cannot counteract the insulin resistance caused by pregnancy. It has a prevalence of approximately 5–15 percent of pregnancies worldwide and is linked to complications such as cesarean section, fetal macrosomia, neonatal hypoglycemia, and a higher risk of type 2 diabetes and cardiovascular disease in the long term (Luo et al., 2026; Wang and Chen, 2026). Preeclampsia is characterized by new-onset hypertension and multi-organ dysfunction after 20 weeks of gestation. It has a prevalence of 2-8% in all pregnancies worldwide and is a major cause of maternal and perinatal mortality (Hughes, 2026; Keitaanpaa, 2026). This disease is closely linked to endothelial dysfunction, systemic inflammation, and abnormal placental development, which result in complications such as fetal growth restriction and preterm birth (Furuta, 2026; Gana, 2026).

An increasing body of evidence suggests that GDM and preeclampsia are not distinct entities but presentations of underlying metabolic and vascular dysfunctions during pregnancy. The co-occurrence of these conditions is caused by shared maternal risk factors such as obesity, advanced maternal age, insulin resistance, and chronic inflammation (Abdi, 2025; Tagami, 2026; Ye, 2025).

At the mechanistic level, GDM and preeclampsia share common biological pathways involving oxidative stress, endothelial dysfunction, angiogenic imbalance, and placental dysfunction, supporting the concept of a unified cardiometabolic and placental disease spectrum (Petkova-Parlapanska, 2025; Zhang, 2025; Chen Z., 2025).

Despite advancements in clinical knowledge, the early diagnosis of GDM and preeclampsia remains challenging. Standard screening methods typically rely on predefined clinical risk factors or diagnostic tests performed during the late second or third trimester, limiting their ability to identify high-risk individuals earlier in pregnancy. Furthermore, the heterogeneous and multifactorial nature of these conditions limits the predictive performance of traditional statistical models, particularly in capturing the complex nonlinear interactions among biological factors (Zheng, 2022; Huang, 2022).

Over the last several years, artificial intelligence (AI), particularly machine learning (ML) and deep learning (DL) techniques, has emerged as a powerful approach for predictive modeling in healthcare. These techniques enable the analysis of high-dimensional, multi-source data, including clinical variables, biochemical markers, electronic health records, and continuous physiological monitoring data (Sharifi-Heris, 2023; Liang, 2023; Ji et al., 2025). AI-based models have demonstrated promising predictive performance in the early prediction of pregnancy complications and show considerable potential to support prenatal risk stratification and personalized maternal care, although further prospective validation and clinical evaluation remain necessary before routine implementation (Zhang J. et al., 2025; Li et al., 2024; Tiruneh et al., 2024).

However, despite the rapid advancement of artificial intelligence (AI)-based prediction models, existing approaches remain largely fragmented and disease-specific, with limited ability to capture the shared cardiometabolic and placental mechanisms underlying gestational diabetes mellitus (GDM) and preeclampsia. Most studies have developed predictive models for each condition independently, despite growing epidemiological and biological evidence indicating that these disorders share common risk factors and overlapping pathophysiological pathways (Ahmed et al., 2025; Liu et al., 2026).

Several systematic reviews have synthesized the application of artificial intelligence for predicting gestational diabetes mellitus (GDM), preeclampsia (PE), or related pregnancy complications (Ahmed et al., 2025; Liu et al., 2026; Ranjbar et al., 2024; Aljameel et al., 2023; Özcan and Peker, 2025; Kokori et al., 2024). However, these reviews differ in their clinical scope, methodological emphasis, and objectives. Table 1 summarizes the principal characteristics of these reviews and positions the present review within the existing literature.

**Table 1:** Comparison of existing systematic reviews on AI-based prediction of gestational diabetes mellitus (GDM) and preeclampsia (PE) with the present review.

Review	Scope	AI	Type	Key contribution	Present review extends by
Ahmed et al. (2025)	GDM + PE	ML	SR	Diagnostic performance of AI models for early prediction of GDM and PE.	47-study synthesis; integration of ML, DL, and multimodal AI; methodological synthesis; evidence-derived conceptual framework.
Liu et al. (2026)	PE	ML	SR + MA	Meta-analysis of machine learning prediction models for preeclampsia.	Integration of GDM evidence; multimodal AI; methodological and implementation synthesis.
Ranjbar et al. (2024)	PE	ML	SR	Systematic synthesis of machine learning models for preeclampsia prediction.	Broader disease scope; DL; multimodal AI; methodological characterization.
Aljameel et al. (2023)	PE	ML/DL	Review	Overview of AI applications for preeclampsia prediction.	Integration of GDM; multimodal AI; structured methodological synthesis.
Özcan and Peker (2025)	PE	ML	SR	Systematic synthesis of machine learning applications for preeclampsia prediction.	Cross-condition synthesis; multimodal AI; evidence-derived conceptual framework.
Kokori et al. (2024)	GDM	ML	Narrative	Review of machine learning algorithms for gestational diabetes prediction.	Inclusion of PE; DL; multimodal AI; implementation considerations.
**Present Review**	**GDM + PE**	**ML + DL + MMAI**	**SR**	**Integrated synthesis of predictive performance, methodological characteristics, implementation challenges, and future research priorities across 47 primary studies**.	**–**

SR, Systematic review; MA, Meta-analysis; ML, Machine learning; DL, Deep learning; MMAI, Multimodal artificial intelligence; GDM, Gestational diabetes mellitus; PE, Preeclampsia. Entries shown in bold represent the proposed framework introduced in the present study.

In addition, many existing AI prediction models rely on single-source or relatively small datasets, limiting their ability to exploit multimodal information that more comprehensively reflects the complex physiological processes of pregnancy (Zaky et al., 2025). Although these models have generally demonstrated encouraging predictive performance, their development has often prioritized accuracy over equally important considerations such as interpretability, biological plausibility, and clinical applicability (Patel, 2023; Chen J., 2025). These limitations have constrained the translation of AI models into routine clinical decision-support systems (CDSS), highlighting the need for more robust, multimodal, explainable, and clinically validated approaches that can support reliable implementation in maternal healthcare (Popova, 2023).

Accordingly, there remains a need for integrated, multimodal, and explainable AI approaches that more comprehensively capture the complex biological interactions underlying gestational diabetes mellitus (GDM) and preeclampsia. Building upon the existing systematic review literature, the present review provides a comprehensive synthesis of AI-based models for the early prediction of GDM and PE by integrating evidence from machine learning, deep learning, and multimodal AI studies. In addition to evaluating methodological quality using PROBAST, the review synthesizes predictive performance, data modalities, predictive features, validation strategies, and implementation challenges across 47 primary studies. Furthermore, it develops an evidence-derived conceptual framework linking AI methodologies with the shared cardiometabolic, vascular, and placental mechanisms underlying these conditions and discusses their implications for future clinical decision-support systems (CDSS) and clinical translation.

To address these gaps systematically, this review aims to: (1) identify and classify machine learning and deep learning models developed for the prediction of gestational diabetes mellitus (GDM) and preeclampsia; (2) evaluate the data modalities and predictive features used in these models; (3) examine their predictive performance and validation strategies; (4) review their potential application in clinical decision-support systems (CDSS); and (5) identify current challenges and propose future research directions for AI-based prediction of GDM and preeclampsia.

The main contributions of this review are as follows:

- A comprehensive synthesis of AI-based predictive models for the early prediction of GDM and preeclampsia.
- An integrated perspective linking AI-based predictive modeling with the shared metabolic, vascular, and placental mechanisms underlying both conditions.
- A systematic comparison of AI models based on data modalities, feature engineering approaches, predictive performance, and validation strategies.
- A critical appraisal of current research gaps, including data fragmentation, limited multimodal integration, model generalizability, interpretability, and challenges associated with clinical translation.
- An evidence-informed conceptual framework together with future research directions emphasizing multimodal learning, explainable AI (XAI), standardized validation, and precision medicine in maternal healthcare.

**Figure 1:**
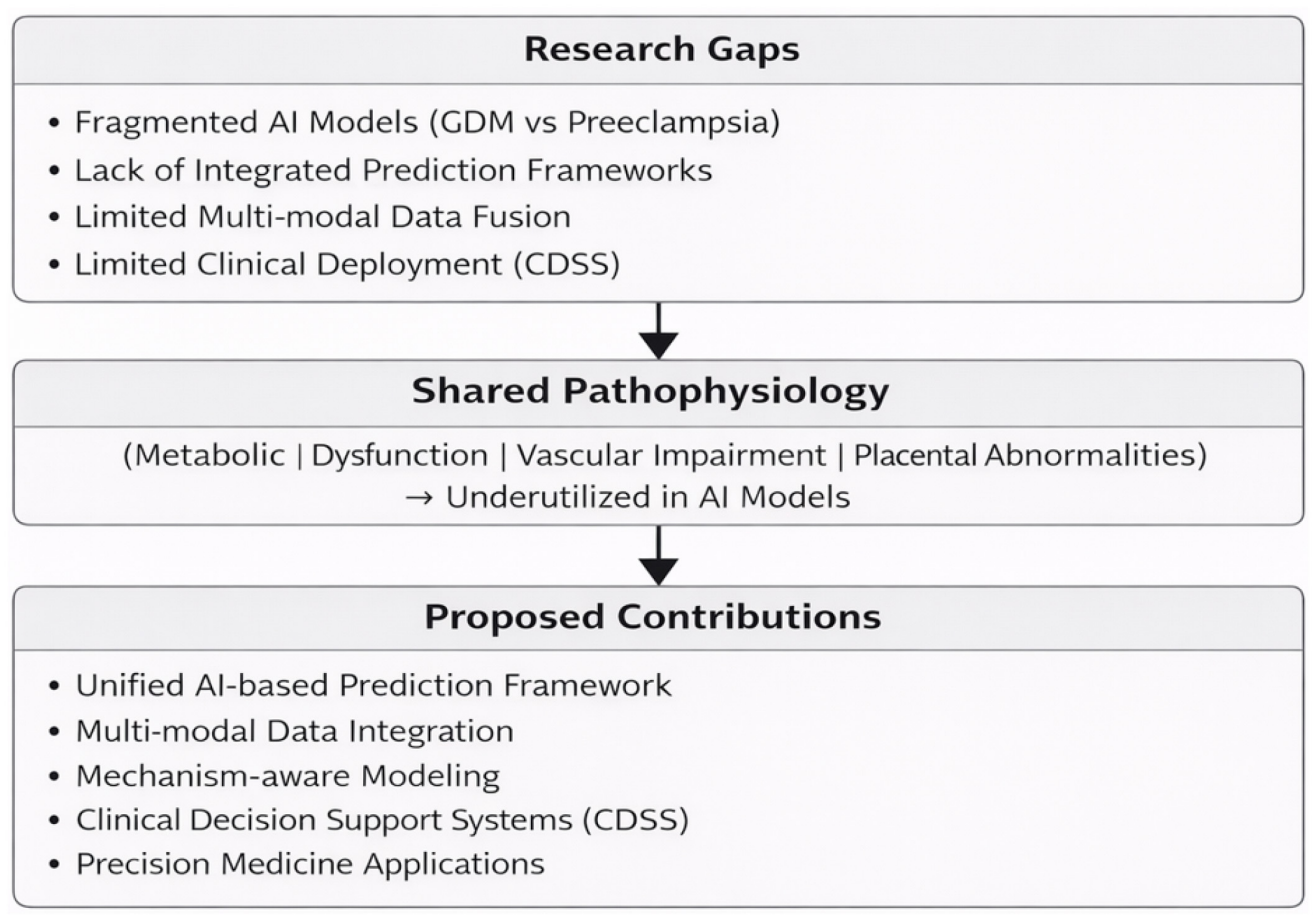
Conceptual illustration of the research gaps addressed and the principal contributions of this review in AI-based prediction of gestational diabetes mellitus and preeclampsia.

The key research gaps and contributions identified in this review are summarized in Figure 1, which illustrates the transition from disease-specific AI prediction models toward an integrated, multimodal, and clinically interpretable conceptual framework for the early prediction of GDM and preeclampsia.

The remainder of this paper is organized as follows. Section 2 presents the background and related work. Section 3 describes the review methodology. Section 4 presents the results, including the analysis and comparison of AI-based prediction models. Section 5 presents an evidence-informed conceptual framework derived from the findings of this review. Section 6 discusses the findings and their clinical and research implications. Finally, Section 7 concludes the paper.

## 2 Existing literature and related work

Over the past few years, there has been growing interest in pregnancy complications because of their high maternal and neonatal morbidity. Gestational diabetes mellitus (GDM) and preeclampsia are two of the most common and clinically significant conditions worldwide (Hughes, 2026). Although historically examined as separate conditions, there is growing evidence of significant overlap in the risk factors, biological pathways, and clinical outcomes of these two conditions, indicating a common underlying cardiometabolic dysfunction (Barrell, 2025; Ye, 2025).

### 2.1 Epidemiology and global burden

GDM is one of the most prevalent metabolic complications of pregnancy, occurring in approximately 5–15 percent of pregnancies worldwide. This increase is attributed to the rising rates of maternal obesity, sedentary lifestyles, and late childbearing (Abdi, 2025; Tagami, 2026). Likewise, preeclampsia occurs in approximately 2-8% of pregnancies and is a major cause of maternal and perinatal morbidity and mortality (Hughes, 2026). Both disorders are linked to poor outcomes, such as preterm birth, fetal growth defects, and cardiovascular disease risk in mothers and offspring in the long term (Luo et al., 2026; Keitaanpaa, 2026; Nguyen, 2024). The increasing worldwide prevalence of these conditions highlights the importance of early diagnosis and preventive strategies.

### 2.2 Shared risk factors and clinical associations

A significant amount of literature has identified common maternal risk factors for GDM and preeclampsia during the first trimester. These include obesity, advanced maternal age, insulin resistance, and metabolic syndrome (Abdi, 2025; Tagami, 2026; Mensah, 2026). A poor diet and physical inactivity are lifestyle-related factors that also contribute to the development of the disease (Sewor, 2024). These common risk profiles confirm the hypothesis that both conditions are caused by underlying metabolic dysregulation and emphasize the significance of early stratification of the disease.

### 2.3 Pathophysiological mechanisms

The pathophysiological processes connecting GDM and preeclampsia are interrelated and multifactorial. Insulin resistance is a key factor in hyperglycemia, endothelial dysfunction, and systemic inflammation. Oxidative stress, immune dysregulation, and abnormal placental development are major contributors (Petkova-Parlapanska, 2025; Furuta, 2026). Trophoblast invasion and decreased placental perfusion result in hypoxia and the release of antiangiogenic factors, which further worsen vascular dysfunction (Bastiansen, 2026). These intersecting processes justify the single model of metabolic, vascular, and placental dysfunction during pregnancy.

### 2.4 Maternal and neonatal outcomes

The presence of GDM and preeclampsia is associated with a high risk of poor maternal and neonatal outcomes (Li, 2025; Umer, 2025). These include cesarean birth, neonatal hypoglycemia, respiratory distress, fetal growth retardation, and preterm birth (Wu and Chen, 2026; Tang W., 2025). Moreover, the two conditions are associated with long-term cardiometabolic risks in mothers and offspring, highlighting their clinical and population health importance (Barrell, 2025; Zhao, 2024).

### 2.5 Long-term health implications

In addition to pregnancy, GDM and preeclampsia are significant predictors of future health. Women with a history of GDM are at a higher risk of developing type 2 diabetes and cardiovascular disease than those without preeclampsia (Schoenaker, 2026), and women with preeclampsia are at risk of developing chronic hypertension and cardiovascular complications (Keitaanpaa, 2026; Yang, 2026). These results support the significance of early diagnosis and lifelong follow-up in patients with MDS.

**Figure 2:**
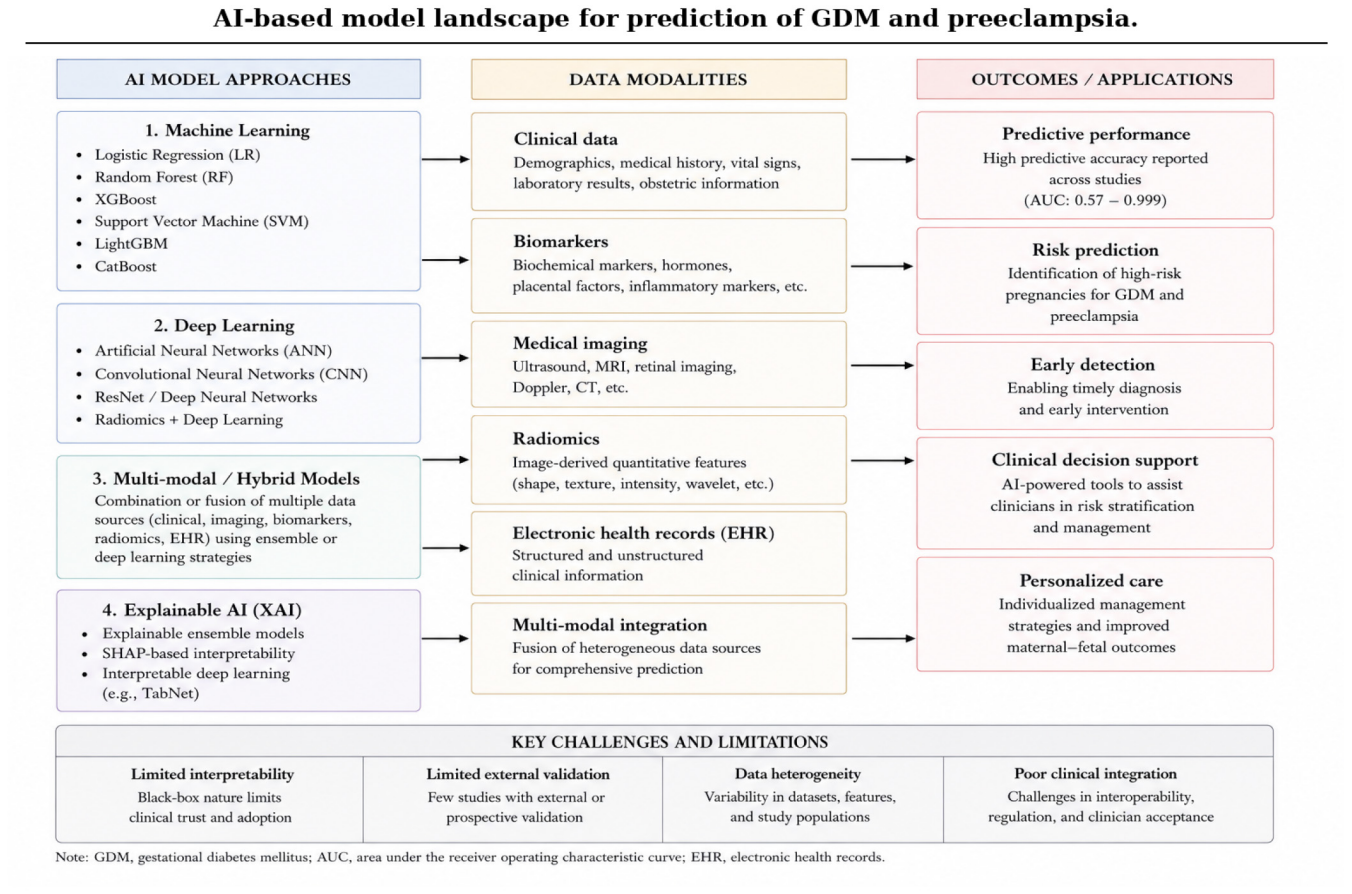
Conceptual overview of the artificial intelligence (AI) landscape for predicting gestational diabetes mellitus (GDM) and preeclampsia (PE). The figure synthesizes the AI approaches identified in the included studies, highlighting model categories, data modalities, clinical applications, predictive performance (reported AUC range: 0.57–0.999), and the principal methodological challenges limiting clinical translation.

Figure 2 provides a synthesized overview of AI-based predictive models in the literature, illustrating the integration of multimodal data sources, model types, predictive performance, and key limitations in supporting early prediction and clinical decision-making.

### 2.6 Artificial intelligence-based prediction of GDM and preeclampsia

Recent advances in artificial intelligence (AI) have transformed predictive modeling in maternal healthcare by enabling the early identification of pregnancies at risk of gestational diabetes mellitus (GDM) and preeclampsia. The growing application of machine learning (ML), deep learning (DL), and multimodal AI has facilitated the development of predictive models using diverse data sources, including clinical characteristics, biochemical biomarkers, medical imaging, radiomics, and electronic health records (Artzi et al., 2020; Zhang, 2026; Zhou H. et al., 2024; Guo et al., 2025; Hassan et al., 2025). Although many of these models demonstrate promising predictive performance, considerable methodological heterogeneity, limited external validation, and inadequate integration into routine clinical practice continue to hinder their widespread implementation (Ahmed et al., 2025; Liu et al., 2026).

Key methodologies, data modalities, and reported performances are summarized in Table 2 and used to structure and critically compare representative models for AI-based predictions.

**Table 2:** Characteristics and quantitative performance of the 47 primary AI prediction studies included in the quantitative evidence synthesis.

#	References	Year	Disease	AI	AI model	Size	Sens.	Spec.	Validation	AUC
1	Guo et al. (2025)	2025	HDP + GDM	Multimodal AI	Clinical + Radiomics + DL Fusion	806	NR	NR	Train/test split	0.963
2	Du et al. (2026)	2026	GDM + PE	Multimodal AI	Deep learning fusion model	177	80.0%	89.58%	Internal validation	0.857
3	Hassan et al. (2025)	2025	GDM	Multimodal AI	Explainable ensemble fusion	NR	97.47%	98.64%	Cross-validation	0.9991
4	Cardenas-Urrelo et al. (2026)	2026	PE	Multimodal AI	Clinical + hemodynamic + biochemical ML fusion	1000	52.0%	94.0%	Internal Validation	0.850
5	Sun et al. (2022)	2022	GDM	Multimodal AI	PFCnet (Gray-scale + microflow image fusion)	718	89%	92%	Independent test set	0.930
6	Sun et al. (2023)	2022	HDP	Multimodal AI	GMNet (gray-scale + microflow image fusion)	654	90.0%	93.5%	Internal validation	0.970
7	Arora et al. (2025)	2025	HDP	Multimodal AI	Placental texture analysis + deep learning fusion	1008	89.1%	91.7%	External validation	0.915
8	Li et al. (2026)	2025	Fetal brain disorders	Multimodal AI	MRI multimodal AI fusion model	806	NR	NR	Clinical validation	0.876
9	Dong et al. (2026)	2026	PE	Multimodal AI	RETINA-TS (Vision transformer + LSTM multimodal fusion)	3910	NR	NR	Internal validation	0.920
10	Liao et al. (2022)	2022	Placental disorders	Multimodal AI	Flow-compensated / non-compensated IVIM MRI fusion	61	NR	NR	Clinical validation	NR
11	Butler et al. (2024)	2024	PE	Deep learning	1D ResNet CNN	1721	NR	NR	External validation	0.850
12	Wang et al. (2024)	2024	GDM	Deep learning	Self-attention CNN	5085	NR	NR	Internal validation	0.9649
13	Zhou H. et al. (2024)	2024	GDM	Deep learning	ResNet50 + radiomics	415	NR	NR	Multicenter validation	0.930
14	Eberhard et al. (2024)	2024	PE	Deep learning	DeepHit survival network	66425	NR	NR	External validation	0.780
15	Narayanan et al. (2026)	2026	GDM	Deep learning	Explainable TabNet	3525	98.91%	NR	Multi-site external validation	NR
16	Wu Y. et al. (2025)	2025	PE	Deep learning	Retinal deep learning model	1812	NR	NR	Prospective validation	0.883
17	Zheng et al. (2025)	2025	PE + FGR	Deep Learning	Deep learning radiomics (MRI-based)	420	NR	NR	Multicenter validation	0.843
18	Zhou T. et al. (2024)	2024	PE	Deep Learning	Inception-ResNet-v2 CNN	1138	72.2%	93.4%	Prospective cohort validation	0.883
19	Kurt et al. (2023)	2023	GDM	Deep learning	Deep neural network with Bayesian optimization	489	95.0%	99.0%	Prospective validation	0.980
20	Bennett et al. (2022)	2022	PE	Deep learning	Cost-sensitive deep neural network	NR	NR	NR	Internal validation	0.765
21	Eberhard et al. (2025)	2025	PE	Deep learning	Longitudinal prediction model	101357	NR	NR	External validation	0.57–0.80
22	Liang et al. (2025)	2025	PE	Deep learning	Deep neural network	200	NR	NR	Retrospective single-center validation	0.838
23	Artzi et al. (2020)	2020	GDM	Machine learning	Gradient boosting	588622	NR	NR	Internal validation	0.854
24	Maric et al. (2020)	2020	PE	Machine learning	Gradient boosting	16370	72.3%	91.2%	Cross-validation	0.89
25	Zhang J. et al. (2025)	2025	GDM	Machine learning	LASSO prediction model	6659	NR	NR	Bootstrap validation	0.736
26	Ji et al. (2025)	2025	GDM	Machine learning	Multilayer perceptron (MLP)	89	NR	NR	Train/test split	0.984
27	Zheng et al. (2022)	2026	GDM	Machine learning	Random forest	NR	NR	NR	Internal validation	0.861
28	Pinto et al. (2023)	2023	GDM	Machine learning	Integrated machine learning framework	394	NR	NR	Internal validation	0.830
29	Du et al. (2022)	2022	GDM	Machine learning	Machine learning prediction model	548	74.5–91.3%	75.7–88.4%	5-fold Cross-validation	0.792
30	Ye et al. (2020)	2020	GDM	Machine learning	Gradient boosting decision tree (GBDT)	22242	90.0%	99.0%	70/30 train/test split	0.740
31	Liu et al. (2020)	2021	GDM	Machine learning	XGBoost	19331	NR	NR	Internal validation	0.742
32	Guo et al. (2020)	2020	GDM + PE + FGR	Machine learning	Logistic regression classifier	2199	NR	NR	Validation	0.720
33	Belsti et al. (2023)	2023	GDM	Machine learning	CatBoost	NR	81%	90%	80/20 train/test split	0.930
34	Xiong et al. (2020)	2020	GDM	Machine learning	LightGBM / SVM	490	88.3%	99.47%	Internal validation	0.942
35	Lu et al. (2021)	2021	GDM	Machine learning	Random forest	1139	65.1%	81.3%	10-fold cross-validation	0.777
36	Hu et al. (2023)	2023	GDM	Machine learning	XGBoost	925	NR	NR	10-fold cross-validation	0.946
37	Syngelaki et al. (2025)	2025	GDM	Machine learning	Logistic regression	41587	87.2%	60.0%	Five-fold cross-validation	0.757
38	Lu et al. (2024)	2024	GDM	Machine learning	Bootstrap + LASSO	60	NR	NR	Bootstrap validation	NR
39	Deng et al. (2024)	2024	GDM	Machine learning	Random forest	600	NR	NR	Internal validation	NR
40	Zhang (2026)	2026	GDM	Machine learning	Support vector machine (SVM)	342	NR	NR	Validation cohort	0.861
41	Scott et al. (2021)	2021	GDM	Machine learning	Metabolomics-based machine learning	NR	NR	NR	Internal validation	NR
42	Hou et al. (2022)	2022	GDM	Machine learning	Random forest	200	NR	NR	validation	0.960
43	Mustafa et al. (2025)	2025	HDP + GDM	Machine learning	AutoML prediction model	33,533	NR	NR	Internal validation	0.89
44	Filippo et al. (2022)	2022	GDM	Machine learning	Machine learning model	73	NR	NR	Internal validation	NR
45	Kaya et al. (2024)	2024	GDM	Machine learning	XGBoost	NR	NR	NR	Internal validation	NR
46	Aminifar (2022)	2022	GDM	Machine learning	Ensemble learning	1012	NR	NR	Internal validation	NR
47	Chan et al. (2023)	2023	GDM	Machine learning	Ensemble machine learning	61	NR	NR	External validation	0.810

#### 2.6.1 Machine learning models

Traditional machine learning (ML) algorithms remain among the most widely adopted approaches for predicting gestational diabetes mellitus (GDM) and preeclampsia because they efficiently analyse structured clinical data while offering relatively high interpretability and low computational cost. Conventional statistical models, particularly logistic regression, continue to serve as important baseline models against which more advanced machine learning algorithms are evaluated (Artzi et al., 2020; Syngelaki et al., 2025). However, their ability to capture the complex nonlinear relationships underlying pregnancy complications is limited.

More advanced tree-based algorithms, including Random Forest, Gradient Boosting Decision Trees (GBDT), LightGBM, CatBoost, and Extreme Gradient Boosting (XGBoost), have consistently demonstrated superior performance by modeling nonlinear interactions and complex feature dependencies. Several studies reported excellent predictive performance, with area under the receiver operating characteristic curve (AUC) values frequently exceeding 0.85, particularly when ensemble learning strategies were employed (Hassan et al., 2025; Hu et al., 2023; Xiong et al., 2020; Belsti et al., 2023; Hou et al., 2022). Similarly, support vector machine (SVM)-based models have demonstrated competitive classification performance, particularly when combined with feature selection and optimized feature engineering (Zhang, 2026).

As shown in Table 2, ML-based approaches consistently achieved strong predictive performance across diverse clinical datasets. Nevertheless, their performance remained highly dependent on data quality, feature selection strategies, and the heterogeneity of patient populations included in model development.

Despite these advances, several limitations continue to hinder the clinical translation of ML-based prediction models. Most studies relied on retrospectively collected data, relatively homogeneous populations, and internal validation, with comparatively few conducting external or prospective validation. Consequently, concerns regarding model generalizability, reproducibility, and implementation in routine clinical practice remain significant challenges (Ahmed et al., 2025; Liu et al., 2026).

#### 2.6.2 Deep learning approaches

Deep learning (DL) approaches have attracted considerable attention because of their ability to automatically learn hierarchical feature representations from high-dimensional and heterogeneous healthcare data. Artificial neural networks (ANNs), convolutional neural networks (CNNs), residual neural networks (ResNet), self-attention networks, and deep radiomics models have been successfully applied to the prediction of gestational diabetes mellitus (GDM) and preeclampsia using clinical, imaging, and multimodal datasets (Wang et al., 2024; Zhou T. et al., 2024; Wu Y. et al., 2025; Zheng et al., 2025; Kurt et al., 2023).

Several studies reported excellent predictive performance, with area under the receiver operating characteristic curve (AUC) values frequently exceeding 0.90, particularly when deep learning architectures were trained using imaging or multimodal datasets (Zhang, 2026; Zhou H. et al., 2024; Guo et al., 2025; Arora et al., 2025; Sun et al., 2022). Deep learning models are especially advantageous for extracting complex nonlinear relationships and high-level features from radiological images and other high-dimensional clinical data, while recurrent and longitudinal architectures further facilitate the modeling of temporal changes throughout pregnancy.

As shown in Table 2, deep learning models generally achieved higher predictive performance than conventional machine learning approaches. However, these gains were accompanied by substantially greater computational requirements, larger training datasets, and reduced model interpretability.

Despite their superior predictive accuracy, several barriers continue to limit the clinical implementation of deep learning models. Their “black-box” nature reduces transparency and clinician confidence in model predictions, while the requirement for large, well-annotated datasets and extensive computational resources restricts their applicability across diverse healthcare settings. Furthermore, relatively few studies performed external or prospective validation, raising concerns regarding the generalizability and real-world deployment of these models (Ahmed et al., 2025; Liu et al., 2026).

#### 2.6.3 Multi-modal and data-driven approaches

A recent trend identified among the included AI studies is the integration of multimodal data sources to improve predictive performance. Multimodal models that combine clinical variables with medical imaging, radiomics, biochemical biomarkers, and deep learning features have consistently demonstrated higher predictive accuracy than models relying on a single data source (Guo et al., 2025; Hassan et al., 2025; Cardenas-Urrelo et al., 2026; Arora et al., 2025).

Multimodal approaches provide a more comprehensive representation of maternal health by integrating complementary clinical, physiological, biochemical, and imaging information. This paradigm is particularly relevant for gestational diabetes mellitus (GDM) and preeclampsia because both disorders arise from complex interactions among metabolic, vascular, inflammatory, and placental mechanisms.

As shown in Table 2, the multimodal AI studies included in this review generally achieved superior predictive performance compared with single-source models. However, relatively few studies implemented multimodal frameworks because of challenges associated with data availability, heterogeneous data integration, interoperability, computational complexity, and limited external validation.

Despite their promising predictive performance, current multimodal AI models remain constrained by fragmented datasets, limited standardization of data collection, and the complexity of integrating heterogeneous clinical and imaging information. Furthermore, relatively few studies incorporated longitudinal physiological measurements or multi-source data collected across pregnancy, highlighting an important opportunity for future research to develop more robust, clinically generalizable, and explainable multimodal prediction models (Guo et al., 2025; Du et al., 2026; Li et al., 2026; Arora et al., 2025).

#### 2.6.4 Explainable AI and clinical interpretability

As AI models become increasingly complex, there has been growing interest in explainable artificial intelligence (XAI) to improve the transparency and clinical interpretability of predictive models. Recent studies have incorporated explainable machine learning frameworks and interpretable deep learning models to identify the clinical variables contributing most to model predictions, thereby enhancing clinician confidence and supporting informed decision-making (Hassan et al., 2025; Narayanan et al., 2026; Ahmed et al., 2025).

Despite these advances, explainability remains underrepresented within the current body of AI literature. Most studies primarily reported predictive performance metrics, such as the area under the receiver operating characteristic curve (AUC), sensitivity, and specificity, with comparatively limited attention given to model transparency, feature attribution, or clinical interpretability. As shown in Table 2, only a small proportion of the included AI studies explicitly incorporated explainable AI techniques, highlighting an important gap between methodological performance and clinical implementation.

Future research should therefore prioritize the development of interpretable or hybrid AI models that combine high predictive accuracy with transparent decision-making, robust external validation, and seamless integration into routine obstetric care (Ahmed et al., 2025; Liu et al., 2026).

#### 2.6.5 Clinical decision support systems

The integration of AI-based prediction models into clinical decision support systems (CDSS) has the potential to facilitate dynamic risk assessment, early identification of high-risk pregnancies, and personalized clinical decision-making. Several studies have demonstrated that machine learning and deep learning models can accurately predict gestational diabetes mellitus (GDM) and preeclampsia using routinely collected clinical, imaging, and laboratory data, thereby providing a strong foundation for future CDSS implementation (Guo et al., 2025; Hassan et al., 2025; Zhang, 2026; Arora et al., 2025).

Despite these promising developments, the translation of AI prediction models into routine clinical workflows remains limited. Most published studies focused primarily on model development and internal validation rather than prospective deployment within real-world healthcare systems. Challenges related to interoperability with electronic health records, regulatory approval, data governance, clinician acceptance, and external validation continue to impede widespread clinical implementation (Ahmed et al., 2025; Liu et al., 2026).

Future research should therefore prioritize prospective multicentre validation, seamless integration with existing healthcare information systems, and user-centered CDSS development to facilitate the safe and effective adoption of AI-assisted prediction models in routine obstetric care.

#### 2.6.6 Limitations in current AI research

Despite the substantial progress made in AI-based prediction of gestational diabetes mellitus (GDM) and preeclampsia, several important methodological and translational limitations remain. First, most studies investigated GDM or preeclampsia as independent conditions, with relatively few developing integrated prediction models that simultaneously account for the shared metabolic, vascular, inflammatory, and placental mechanisms underlying both disorders (Ahmed et al., 2025; Liu et al., 2026).

Second, considerable heterogeneity exists across the included studies with respect to study populations, sample sizes, predictor variables, feature selection techniques, AI algorithms, validation strategies, and performance metrics. This methodological variability limits direct comparison between studies and hinders the development of standardized and clinically generalizable prediction models. Furthermore, although many studies reported excellent internal performance, comparatively few performed external or prospective validation, raising concerns regarding model robustness, reproducibility, and generalizability across diverse healthcare settings (Ahmed et al., 2025; Liu et al., 2026).

Third, the comparative analysis presented in Table 2 demonstrates that relatively few studies combined multimodal data integration, explainable AI techniques, and rigorous external validation within a single predictive framework. Consequently, many existing models remain difficult to interpret, challenging to implement within routine clinical workflows, and insufficiently validated for widespread clinical adoption.

Finally, only a limited number of studies explicitly integrated biological knowledge with data-driven modeling by combining clinical variables with imaging, radiomics, placental characteristics, or biochemical biomarkers within multimodal AI frameworks (Guo et al., 2025; Cardenas-Urrelo et al., 2026; Arora et al., 2025). Bridging mechanistic understanding with advanced AI modeling represents an important opportunity for developing clinically meaningful, biologically informed, and more generalizable prediction models capable of supporting precision obstetric care.

In general, the literature shows a clear evolution of the traditional single-variable models to integrated multimodal artificial intelligence models; however, there are still limitations associated with interpretability, generalizability, and clinical implementation, which indicate that there are several gaps that need to be filled to further develop the field.

Although significant advances have been made, several gaps remain in the literature on gestational diabetes mellitus (GDM) and preeclampsia. First, most studies have examined these conditions separately, and few have examined their mutual pathophysiological interactions in a single predictive model (Ahmed et al., 2025). This fragmented methodology limits the development of comprehensive models that can explain the intricate interactions between metabolic, vascular, and placental dysfunctions.

Second, there was a high level of heterogeneity among the studies in terms of the diagnostic criteria, study design, population characteristics, and reported outcomes. This heterogeneity hinders the comparability of studies and the development of standardized and generalizable predictive models (Liu et al., 2026).

Artificial intelligence can also identify other issues. Most predictive models are constructed from a small number of clinical variables but seldom include other relevant data sources (e.g., biomarkers, electronic health records, continuous monitoring data, and omics data) into the model (Zaky et al., 2025). Moreover, the majority of machine learning studies mainly focus on the prediction of either gestational diabetes mellitus (GDM) or preeclampsia individually, even though both conditions share many common underlying biological and clinical risk mechanisms (Ahmed et al., 2025).

Another important problem is that there is no external validation of the current AI models. Models tend to perform well only in the populations in which they were developed, raising concerns of bias, poor generalizability, and limited clinical applicability across healthcare settings (Patel, 2023). However, the non-use of explainable artificial intelligence techniques decreases the transparency of the models and hinders clinicians trust and interpretation of the prediction results (Chen J., 2025). They have the potential of performance prediction, but very few models have been translated successfully into clinical decision support systems for real-world healthcare practice (Popova, 2023).

Insulin resistance, endothelial dysfunction and placental abnormalities have been explored in detail, however, few studies have integrated this biological knowledge with data driven predictive modeling approaches (Ji et al., 2025). This leaves a large gap between mechanistic understanding and computational prediction in this field.

Overcoming these limitations requires a multidimensional and holistic solution, integrating the knowledge of pathophysiology with state-of-the-art artificial intelligence techniques. This would facilitate the development of powerful, interpretable, and clinically meaningful predictive models for early risk stratification and improve maternal health care outcomes.

These gaps highlight the need for comprehensive, interpretable, and clinically integrated AI systems that can jointly model gestational diabetes mellitus and preeclampsia in a precision medicine paradigm. These findings support the relevance of early detection and long-term follow-up in these patients. Figure 2 illustrates a conceptual map of the integration of multimodal data sources and artificial intelligence models in the current literature for early prediction and clinical decision-making.

## 3 Methodology

This study presents a systematic review of artificial intelligence-based predictive modeling methods for the early detection of gestational diabetes mellitus (GDM) and preeclampsia, following the PRISMA 2020 reporting guidelines (Page et al., 2021).

### 3.1 Protocol and reporting standards

This review aimed to identify, appraise, and synthesize the current literature on AI-driven models for GDM and preeclampsia, with a focus on ML, DL, and CDSS for maternal risk stratification.

The methodology was guided by the Preferred Reporting Items for Systematic Reviews and Meta-Analyses (PRISMA) 2020 framework to ensure methodological transparency, reproducibility and rigor. No formal protocol was registered in PROSPERO. However, a systematic and defined process was followed. Research objectives, eligibility criteria, search strategies and data extraction procedures were well defined.

The analysis also included the prediction performance, data modalities, validation strategies, interpretability, and clinical utility of existing AI models. The framework was developed based on population (pregnant women), intervention (AI-based predictive models), comparison (traditional or non-AI based approaches, when applicable), and outcomes (predictive performance and clinical applicability).

### 3.2 Search strategy

A systematic literature search was conducted using two major bibliographic databases, namely Scopus and Web of Science. The electronic database searches were last updated on 2 July, 2026. These databases were selected because they provide comprehensive coverage of high-quality, peer-reviewed literature across the fields of medicine, artificial intelligence, biomedical engineering, and health informatics. The electronic database search identified a total of 1,638 records, comprising 700 records retrieved from Scopus and 938 records retrieved from Web of Science. To maximize the completeness of the review, the electronic database search was supplemented by manual reference-list screening and citation tracking, which identified an additional 112 records, resulting in a total of 1,750 records for screening.

The search was limited to articles published between 2020 and 2026 and focused on recent advances in artificial intelligence for the prediction of gestational diabetes mellitus (GDM) and preeclampsia (PE). Eligible studies were required to be peer-reviewed, published in English, involve human subjects, and report original research. The search strategy was designed to be reproducible by using predefined keywords, Boolean operators, and standardized search strings that were consistently applied across both databases.

The search strategy was developed using Medical Subject Headings (MeSH), where applicable, together with free-text keywords related to pregnancy complications and artificial intelligence techniques.

- “gestational diabetes mellitus” OR “GDM” OR “preeclampsia” OR “hypertensive disorders of pregnancy”
- “machine learning” OR “artificial intelligence” OR “deep learning”
- “prediction” OR “early detection”
- “clinical decision support systems” OR “CDSS”

Database-specific search queries were constructed using Boolean operators. An example of the search strategy is presented below:


  (‘‘gestational diabetes mellitus'' OR ‘‘GDM''
  OR ‘‘preeclampsia'' OR ‘‘hypertensive disorders
  of pregnancy'')
  AND
  (‘‘machine learning'' OR ‘‘0Multimodal artificial
  intelligence''1 OR ‘‘2deep learning''3)
  AND
  (‘‘4prediction''5 OR ‘‘6early detection''7)


In addition to the electronic database search, manual reference-list screening and citation tracking were performed to identify additional eligible studies that were not retrieved during the initial database search.

### 3.3 Study eligibility criteria

Studies were selected based on predefined inclusion and exclusion criteria aligned with the objective of evaluating AI-based predictive models.

#### 3.3.1 Inclusion criteria

- Studies developing or validating AI, ML, or DL models for prediction of GDM, preeclampsia, or both
- Studies involving clinical decision support systems (CDSS)
- Studies using clinical, biomarker, imaging, or multi-modal datasets
- Peer-reviewed articles published in English (2020–2026)
- Studies reporting model performance metrics such as AUC, accuracy, sensitivity, and specificity

#### 3.3.2 Exclusion criteria

- Studies not involving AI or predictive modeling
- Purely descriptive clinical or epidemiological studies without computational modeling
- Case reports, editorials, and conference abstracts without full text
- Non-human studies
- Studies lacking sufficient methodological or performance details

### 3.4 Study selection process

In the identification stage, 1,750 records were screened for eligibility. Among them, 1,638 records were found in the chosen databases (Scopus and Web of Science), and 112 more records were located through reference-list screening and citation tracking. After eliminating 268 duplicate records, 1,482 unique studies were analyzed in this review.

In the screening phase, 1,482 records were screened based on their title and abstract. Two independent reviewers performed title/abstract screenings and full-text assessments. Any differences were addressed through discussion and consensus to achieve consistency and reduce the selection bias. Consequently, 903 studies were eliminated because they were irrelevant to the study area, did not align with the AI-based prediction of GDM and preeclampsia, or had inadequate methodological descriptions.

In the eligibility stage, 579 full-text articles were assessed for eligibility. Among them, 459 studies were filtered out due to conceptual or methodological mismatch, irrelevance to AI-based prediction of gestational diabetes mellitus and preeclampsia, or inadequate reporting of model development or performance.

Lastly, 120 studies were included in the qualitative synthesis as they met the inclusion criteria. The literature summary in Table 2 shows a subset of 47 representative studies, and the full list is provided in the Supplementary Table S1.

**Figure 3:**
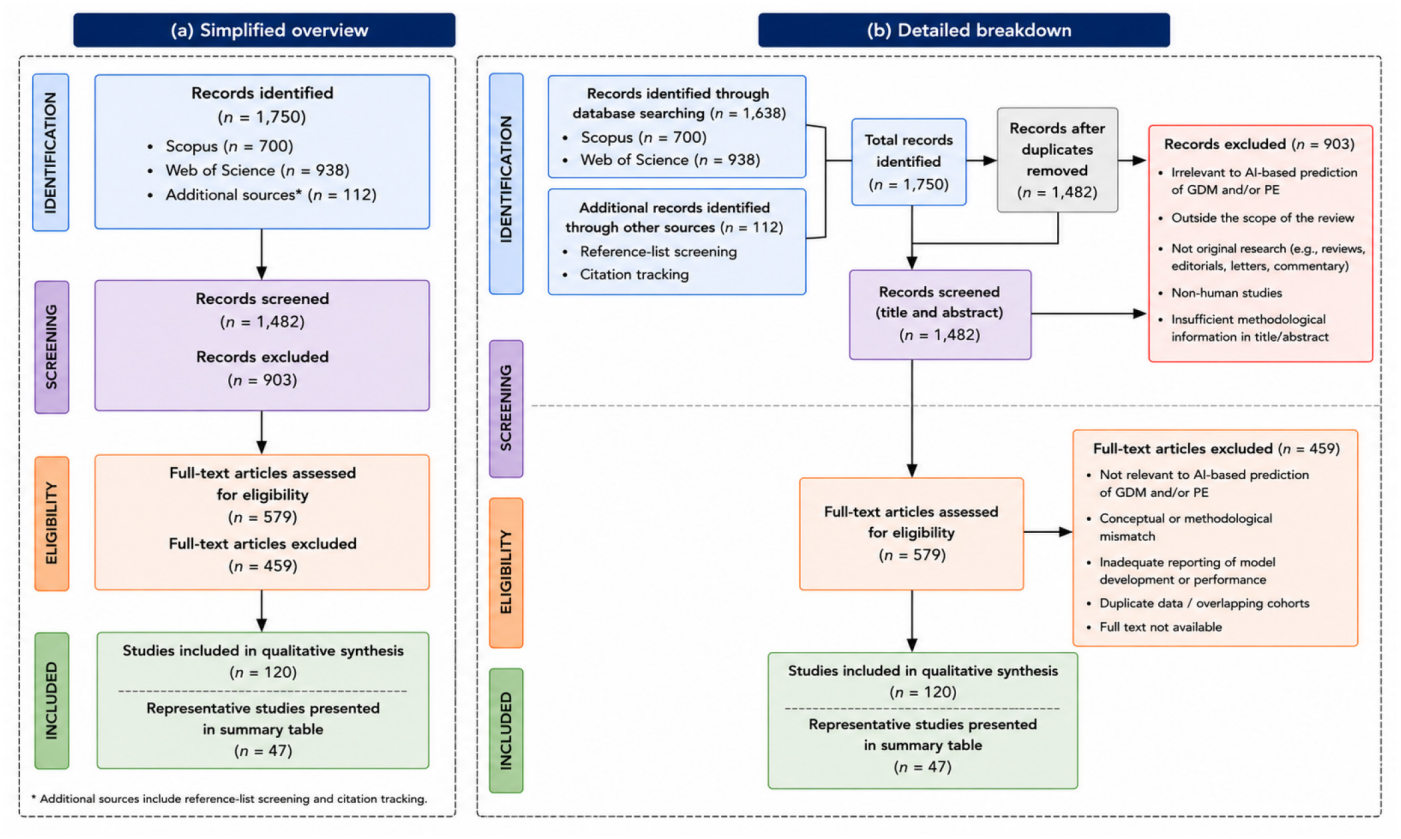
PRISMA 2020 flow diagrams illustrating the study selection process: **(a)** simplified overview of the identification, screening, eligibility, and inclusion stages; and **(b)** detailed breakdown of the study selection process, including the reasons for study exclusion.

Figure 3 shows the PRISMA 2020 flow diagram of the study selection process, including a summary and breakdown of study identification, screening, eligibility, and inclusion.

### 3.5 Data extraction

Data extraction was independently conducted by two reviewers using a structured framework specifically developed for studies involving artificial intelligence (AI)-based predictive models. A standardized data extraction form was used to ensure consistency and completeness throughout the review process. The extracted information included:

- Author(s) and publication year;
- Study design and dataset characteristics;
- Type of AI model (e.g., logistic regression, random forest, support vector machine, deep learning, or multimodal AI);
- Input features, including clinical variables, biomarkers, imaging, and multimodal data;
- Predictive performance metrics, including the area under the receiver operating characteristic curve (AUC), accuracy, sensitivity, specificity, and other reported evaluation measures;
- Validation strategies, including internal validation, external validation, prospective validation, multicentre validation, and cross-validation;
- Use of explainable artificial intelligence (XAI) techniques, where applicable; and
- Clinical applicability and potential integration into clinical decision support systems (CDSS).

Data extraction was independently performed by two reviewers, and cross-verification was undertaken to ensure the accuracy and consistency of the extracted information. Any disagreements were resolved through discussion and consensus. In addition to extracting study characteristics and model performance, information relevant to methodological quality, including validation procedures, dataset characteristics, reporting transparency, and model reproducibility, was also collected to support the subsequent quality assessment.

### 3.6 Evidence classification

To improve the transparency of the evidence synthesis, the included studies were classified according to their primary contribution to the review. Four evidence categories were defined: (i) Primary AI Prediction Studies, (ii) AI Supporting and Translational Studies, (iii) Clinical, Epidemiological and Outcome Studies, and (iv) Mechanistic and Biomarker Studies. This classification framework guided both the quantitative and narrative syntheses. The resulting distribution of studies across these evidence categories is presented in Table 3, while the detailed classification of each included study is provided in Supplementary Table S1.

**Table 3:** Distribution of the included studies across the evidence categories used in the systematic review.

Evidence category	Number of studies
Category I—Primary AI prediction studies	65
Category II—AI supporting & Translational studies	9
Category III—Clinical, epidemiological & Outcome studies	24
Category IV—Mechanistic & Biomarker studies	22
**Total**	**120**

### 3.7 Quality assessment

The methodological quality of the primary artificial intelligence (AI) prediction studies was evaluated using an AI-specific assessment framework designed to assess methodological rigor, computational robustness, reproducibility, and clinical applicability of predictive modeling studies. Unlike conventional appraisal tools developed primarily for observational clinical studies, this framework was designed to evaluate methodological characteristics that are particularly relevant to AI-based prediction models, including model development, validation strategies, reporting transparency, feature engineering, and model interpretability.

The methodological assessment considered the following domains:

- Validation strategy (internal, external, multicentre, prospective, or cross-validation);
- Risk of model overfitting;
- Dataset size and representativeness;
- Feature selection and feature engineering methodology;
- Transparency and completeness of methodological reporting; and
- Use of explainable artificial intelligence (XAI) techniques to enhance model interpretability and clinical applicability.

The methodological quality assessment was performed independently by two reviewers, with disagreements resolved through discussion and consensus. The aggregated findings are summarized in Table 4. This assessment complements the formal risk-of-bias evaluation performed using the Prediction model Risk Of Bias ASsessment Tool (PROBAST), a validated instrument specifically developed for diagnostic and prognostic prediction model studies (Wolff et al., 2019; Moons et al., 2019).

**Table 4:** Summary of the methodological quality assessment of the included artificial intelligence studies.

Assessment criterion	Observation
Validation strategy	External, multicentre, multi-site external, prospective, prospective cohort or clinical validation (*n* = 17); Internal or training/validation (*n* = 14); Cross-validation, bootstrap validation, independent test set, or train–test split (*n* = 16).
Dataset representativeness	All 47 studies used clinical datasets. Most studies were hospital-based, while multicentre cohorts were reported in studies employing multicentre or multi-site validation (*n* = 3).
Feature selection and engineering	The majority of studies employed systematic feature engineering using radiomics, statistical feature selection, machine learning algorithms, deep learning feature extraction, or multimodal feature fusion.
Risk of overfitting	Thirty-three studies (70.2%) employed model validation procedures, including external, internal, prospective, multicentre, or clinical validation, whereas 14 studies (29.8%) primarily relied on resampling approaches such as cross-validation, bootstrap validation, or train–test split. Overall, the widespread use of validation procedures reduced, but did not eliminate, the potential risk of model overfitting.
Reporting transparency	Most studies adequately reported the AI model, validation strategy, dataset characteristics, and predictive performance metrics, thereby supporting methodological reproducibility and comparison across studies.
Explainable artificial intelligence	Explicit explainable AI techniques (e.g., SHAP, Grad-CAM, attention mechanisms, interpretable ensembles, or explainable neural networks) were reported in 4 studies (8.5%), while the remaining studies primarily focused on predictive performance.
Overall methodological quality	Overall, the evidence indicated moderate-to-high methodological quality across the included studies. Higher-quality studies were generally characterized by robust validation strategies, transparent methodological reporting, clinically representative datasets, and, where applicable, the incorporation of explainable AI techniques. Nevertheless, broader multicentre external validation and prospective clinical implementation remain important priorities for future research.

### 3.8 Risk of bias assessment

In addition to the AI-specific methodological quality assessment, the risk of bias of the primary AI prediction studies was evaluated using the Prediction model Risk Of Bias ASsessment Tool (PROBAST), a validated instrument specifically developed for assessing the risk of bias and applicability of diagnostic and prognostic prediction model studies (Wolff et al., 2019; Moons et al., 2019). PROBAST is recommended for systematic reviews of clinical prediction models and evaluates methodological quality across four domains: participants, predictors, outcome, and analysis.

The PROBAST assessment was applied only to the primary AI prediction studies because these studies developed or validated diagnostic or prognostic prediction models. Supporting clinical, epidemiological, mechanistic, biomarker, and review studies were not assessed using PROBAST because they did not involve the development or validation of prediction models.

Each study was independently evaluated using the PROBAST signaling questions, and each domain was assigned a judgement of low, high, or unclear risk of bias. Particular attention was given to issues commonly encountered in AI-based prediction studies, including participant selection, predictor definition, outcome ascertainment, sample size, model overfitting, handling of missing data, validation strategy, calibration, and reporting transparency. Disagreements between reviewers were resolved through discussion and consensus.

**Figure 4:**
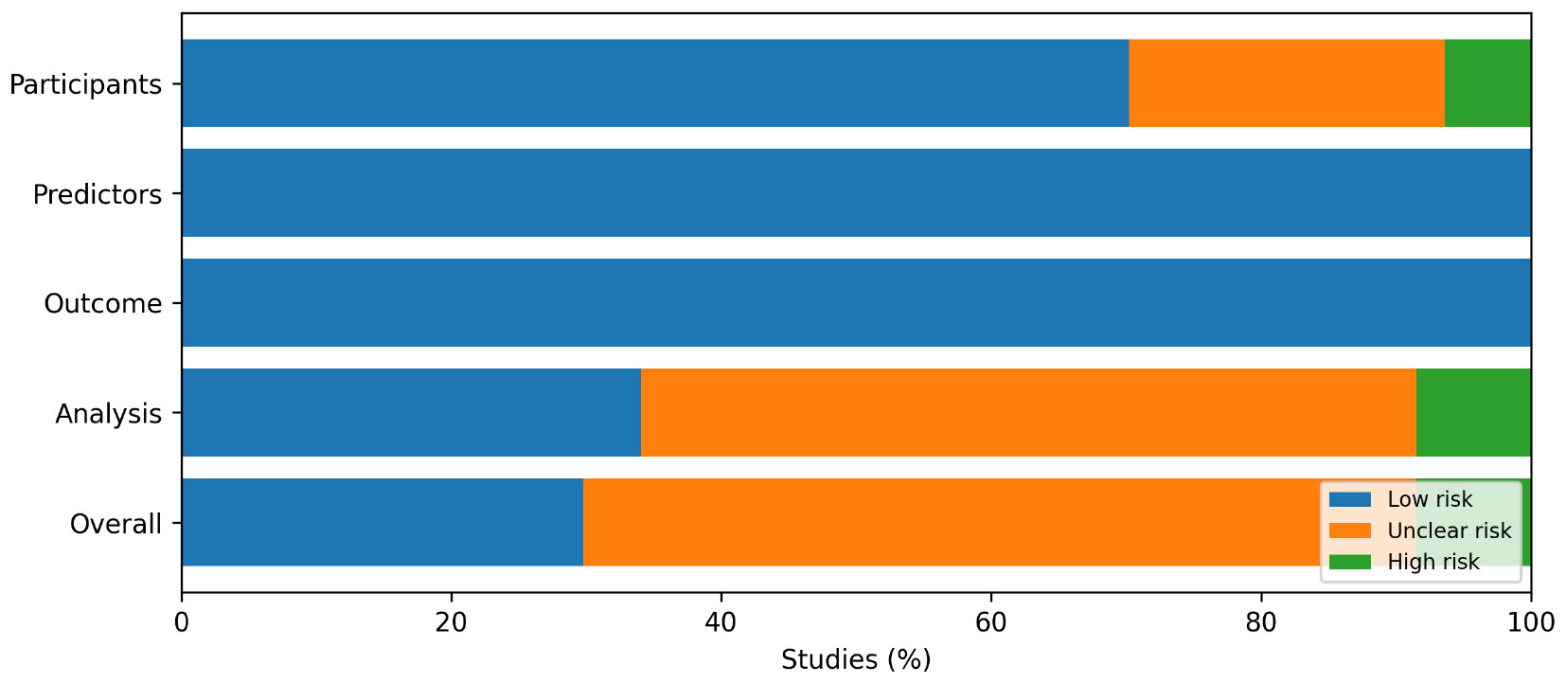
Summary of the PROBAST risk-of-bias assessment across the 47 primary AI prediction studies. Green indicates low risk of bias, yellow indicates unclear risk of bias, and red indicates high risk of bias for each PROBAST domain (Participants, Predictors, Outcome, and Analysis).

The results of the PROBAST assessment are summarized in Table 5 and Figure 4, and are discussed in the Results section.

**Table 5:** Summary of the PROBAST risk-of-bias assessment of the 47 primary AI prediction studies.

PROBAST domain	Low risk	Unclear risk	High risk
Participants	33 (70.2%)	11 (23.4%)	3 (6.4%)
Predictors	47 (100.0%)	0 (0.0%)	0 (0.0%)
Outcome	47 (100.0%)	0 (0.0%)	0 (0.0%)
Analysis	16 (34.0%)	27 (57.4%)	4 (8.5%)
Overall Risk of Bias	14 (29.8%)	29 (61.7%)	4 (8.5%)

### 3.9 Data synthesis and analysis

Data synthesis and analysis will be performed using the following subheadings: The heterogeneity of the study designs, datasets, and reported outcomes led to the adoption of a narrative synthesis approach in this review. This is a suitable method because the data modalities, model architectures, and evaluation metrics vary among AI-based studies. The models were compared based on standardized performance metrics, validation strategies, data modalities, and clinical applicability. The methodology has limitations, such as the possibility of publication bias, the limitation to English-language studies, and heterogeneity in reporting standards in the included studies.

The studies included were categorized into thematic groups according to their methodological focus.

- AI models for gestational diabetes mellitus prediction.
- AI models for preeclampsia prediction.
- Multi-modal and integrated prediction models.
- Clinical decision support systems.

A comparative study was conducted to determine the trends in the model performance, data modalities, and validation strategies. Special consideration was given to the main AI-related aspects, such as model generalizability, robustness, and clinical relevance. A quantitative meta-analysis was not conducted because of high methodological heterogeneity, such as variations in the study populations, feature sets, model types, and performance metric reporting. Attempts were made to assess the reproducibility of the AI models based on the accessibility of methodological information and validation methods reported in the studies.

The results are presented across major thematic areas, including shared risk factors, pathophysiological mechanisms, maternal and neonatal outcomes, and AI-based predictive modeling. A detailed summary of all the included studies is provided in Supplementary Table S1. The review process was designed to ensure transparency and reproducibility through clearly defined inclusion criteria, structured data extraction, and a systematic analysis. As this study was based exclusively on previously published data, ethical approval was not required.

### 3.10 Limitations of the review methodology

This review had some methodological limitations. First, the use of English-language publications only could have led to a language bias. Second, there may have been potential publication bias because unpublished studies and gray literature were not included. Third, the comparability and generalizability of the findings may have been influenced by the high heterogeneity of the study design, datasets, feature selection methods, and reporting standards among the included studies.

Moreover, differences in the diagnostic criteria and outcome definitions of gestational diabetes mellitus and preeclampsia can also be a limitation of cross-study comparisons. Despite these shortcomings, methodological rigor was achieved using predetermined inclusion criteria, systematic data extraction, and systematic analysis, which facilitated a systematic, transparent, and reproducible synthesis of the existing evidence on AI-based predictive models of gestational diabetes mellitus and preeclampsia.

## 4 Results and synthesis

This section summarizes the studies included, with an emphasis on the developments in artificial intelligence (AI)-based predictive modeling and the epidemiological association between gestational diabetes mellitus (GDM) and preeclampsia. The results are presented in a way that emphasizes the performance of the model, data integration, validation, and clinical applicability, as well as important clinical and pathophysiological insights.

### 4.1 Study selection and characteristics

A total of 120 studies were eligible and included in the qualitative synthesis (Supplementary Table S1), as shown in Figure 3. The included studies were published between 2020 and 2026 and represented diverse geographical regions, including Asia, Europe, and North America, with the majority originating from Asian populations.

**Figure 5:**
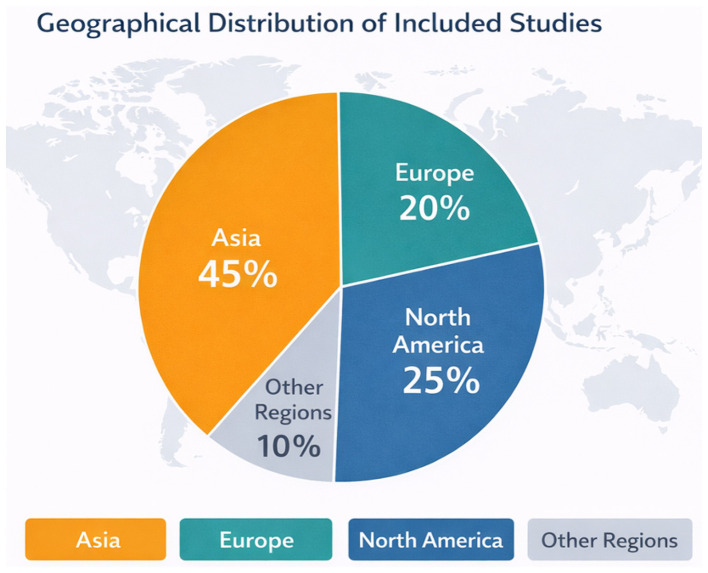
Geographical distribution of included studies on AI-based prediction of gestational diabetes mellitus and preeclampsia. The majority of studies were conducted in Asia, followed by Europe and North America, with fewer studies from other regions.

**Figure 6:**
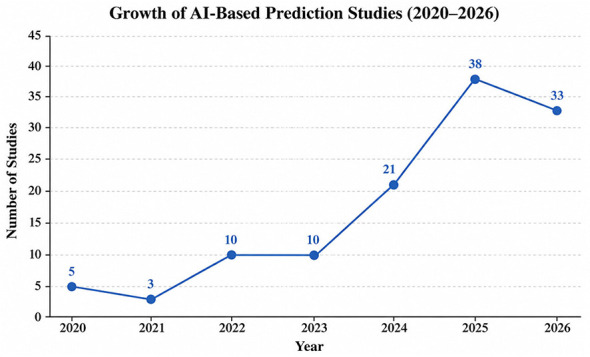
Year-wise distribution of the included studies (*n* = 120) on AI-based prediction of gestational diabetes mellitus and preeclampsia from 2020 to 2026. The figure demonstrates a steady increase in the number of studies over time within the selected literature.

Table 6 summarizes the distribution of studies by publication year, demonstrating a marked increase in research activity over the review period. The geographical distribution of the included studies is presented in Figure 5. The temporal trend in the number of included studies is illustrated in Figure 6.

**Table 6:** Distribution of included studies by publication year.

Year	2026	2025	2024	2023	2022	2021	2020	Total
Count	33	38	21	10	10	3	5	**120**

Overall, the evidence base comprised observational studies, including cohort, case-control, and cross-sectional studies, together with systematic reviews, meta-analyses, and an increasing number of artificial intelligence (AI)-based predictive studies. Collectively, these studies examined epidemiological relationships, shared risk factors, pathophysiological mechanisms, clinical outcomes, and predictive modeling strategies for gestational diabetes mellitus (GDM) and preeclampsia (Ahmed et al., 2025; Liu et al., 2026).

The final evidence base comprised 120 studies, which were classified into four complementary evidence domains to facilitate the synthesis of heterogeneous literature (Table 5). Primary AI Prediction Studies constituted the largest category (*n* = 65), followed by Clinical, Epidemiological and Outcome Studies (*n* = 24), Mechanistic and Biomarker Studies (*n* = 22), and AI Supporting and Translational Studies (*n* = 9).

**Figure 7:**
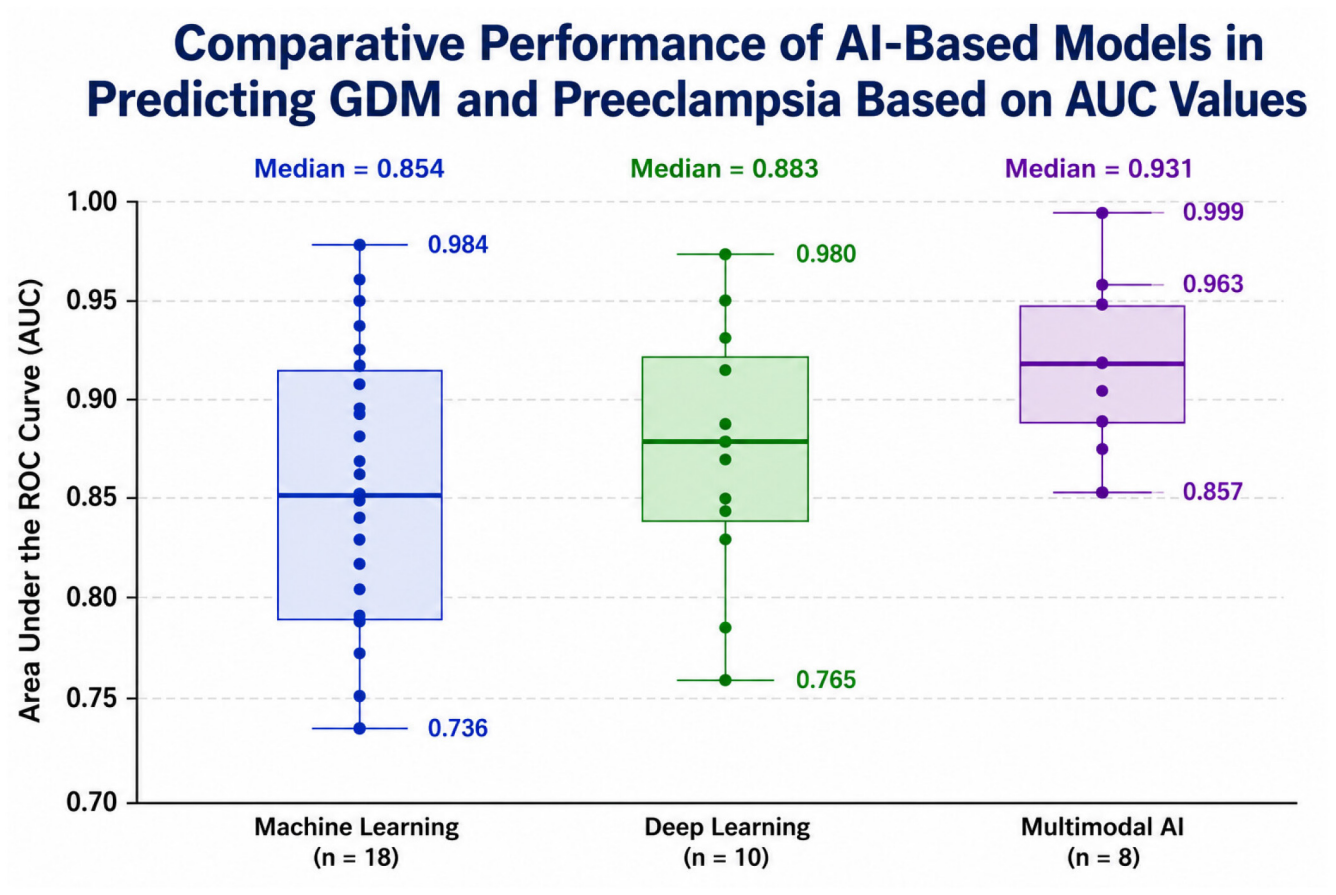
Distribution of reported area under the receiver operating characteristic curve (AUC) values across Machine Learning (ML), Deep Learning (DL), and Multimodal AI models included in this systematic review. The box plots summarize the median, interquartile range (IQR), and minimum–maximum AUC values, while each point represents an individual study. Only studies reporting a single numerical AUC value for the proposed model were included in the quantitative comparison (ML, *n* = 18; DL, *n* = 10; Multimodal AI, *n* = 8). Studies reporting no numerical AUC values (NR) or only AUC ranges were excluded from this analysis but were retained in the qualitative synthesis (Table 2).

This evidence classification reflects the multidisciplinary nature of AI-based prediction research, in which predictive model development is supported by complementary methodological, clinical, epidemiological, and biological evidence. Accordingly, although all 120 studies contributed to the overall evidence synthesis, the comparative analysis of predictive performance (Table 2) and the quantitative AUC synthesis (Figure 7) were restricted to the subset of primary AI prediction studies that reported sufficient methodological and performance information.

### 4.2 Methodological quality and risk-of-bias assessment using PROBAST

The methodological risk of bias of the 47 primary AI prediction studies was evaluated using the Prediction model Risk Of Bias ASsessment Tool (PROBAST). Overall, most studies demonstrated a low risk of bias in the Participants, Predictors, and Outcome domains, whereas the Analysis domain represented the principal source of methodological concern owing to the predominant use of internal validation strategies without independent external validation. The domain-level summary of the PROBAST assessment is presented in Table 5 and Figure 4, while the complete study-level assessments are provided in Supplementary Table S2.

The domain-level distribution of the PROBAST assessment is further illustrated in Figure 4.

Overall, the PROBAST assessment indicated that most primary AI prediction studies presented a low risk of bias in the Participants, Predictors, and Outcome domains, reflecting the widespread use of clinically representative datasets, objectively measured predictors, and standardized diagnostic criteria for gestational diabetes mellitus and preeclampsia. The Analysis domain demonstrated the greatest methodological concern because many studies relied primarily on internal validation, cross-validation, bootstrap validation, or train–test split approaches without independent external validation or comprehensive calibration assessment. Consequently, the principal source of potential bias was related to model development and analytical validation rather than participant selection or outcome ascertainment. Detailed study-level PROBAST assessments are provided in Supplementary Table S2.

### 4.3 AI-based predictive modeling: performance, data integration, and clinical translation

Artificial intelligence (AI)-based predictive modeling constituted the largest evidence domain identified in this systematic review. The 47 primary AI prediction studies included in the quantitative evidence synthesis (Table 2) evaluated a diverse range of machine learning (ML), deep learning (DL), and multimodal AI approaches for predicting gestational diabetes mellitus (GDM), preeclampsia (PE), or both conditions. Commonly employed algorithms included logistic regression, support vector machines (SVM), random forests, gradient boosting algorithms (e.g., XGBoost), artificial neural networks (ANN), convolutional neural networks (CNN), and hybrid ensemble learning models (Zheng, 2022; Huang, 2022; Hassan et al., 2025).

The evolution of AI methodologies across the included studies, illustrated in Figure 2, demonstrates a clear progression from conventional machine learning algorithms toward deep learning and multimodal AI frameworks capable of integrating heterogeneous clinical, biochemical, imaging, radiomics, and omics data to improve predictive performance and clinical applicability (Zaky et al., 2025; Ji et al., 2025).

Importantly, the quantitative evidence synthesis identified only four(4) of the 47 primary AI prediction studies as developing integrated prediction models for gestational diabetes mellitus (GDM) together with preeclampsia (PE) or related hypertensive disorders of pregnancy (HDP), whereas the remaining studies focused on prediction of a single pregnancy complication. These joint-prediction studies predominantly employed multimodal or integrated artificial intelligence approaches that combined complementary clinical, imaging, biomarker, and radiomics data. Their limited number demonstrates that integrated prediction of GDM and hypertensive pregnancy disorders remains an emerging research area and represents one of the principal methodological gaps identified in this review.

Overall, the AI prediction studies demonstrated good discriminatory performance, with most reported area under the receiver operating characteristic curve (AUC) values exceeding 0.80. Conventional machine learning models, including logistic regression, random forests, support vector machines, gradient boosting, CatBoost, and XGBoost, remained the most frequently implemented approaches because of their robustness, interpretability, and suitability for structured clinical datasets (Zhang J. et al., 2025). However, recent studies increasingly adopted deep learning and multimodal AI architectures capable of analysing complex imaging, laboratory, and multimodal datasets, frequently achieving AUC values exceeding 0.90 when trained using large and heterogeneous datasets (Sharifi-Heris, 2023; Liang, 2023; Li et al., 2024; Zhou T. et al., 2024; Wu Y. et al., 2025; Adil et al., 2025).

The comparative performance of the extracted AI models is summarized in Figure 7. Overall, multimodal AI and deep learning models demonstrated higher median AUC values than conventional machine learning approaches, reflecting the advantages of integrating complementary clinical, biochemical, imaging, and longitudinal data to capture the complex pathophysiological interactions underlying pregnancy complications. Nevertheless, substantial variability remained across studies because of differences in dataset characteristics, feature selection strategies, outcome definitions, and validation methodologies. Consequently, although multimodal AI represents the current direction of AI-based prediction research, broader multicentre datasets, standardized reporting frameworks, external validation, and prospective clinical evaluation remain essential before these models can be routinely implemented in clinical practice.

As shown in Figure 7, the multimodal and deep learning models consistently achieved higher AUC values, reflecting their ability to capture complex high-dimensional relationships in heterogeneous datasets (Zhou T. et al., 2024; Lei et al., 2026). Studies integrating continuous monitoring data and multi-omics features have demonstrated the highest predictive accuracy, highlighting the importance of data richness in model performance. The differences in the reported results were due to the variability in the dataset size, feature selection, and validation strategies.

Model performance depends on the type of input data and the degree of data integration. Models based solely on clinical variables (maternal age, BMI, blood pressure) had moderate predictive accuracy, while models based on biochemical markers, electronic health records (EHR), continuous glucose monitoring (CGM), and multi-omics data achieved much better performance (Ballard et al., 2024). The consistently superior performance of multimodal models over single-source models (Chen Z., 2025) shows the value of combining metabolic, vascular and inflammatory information. Table 7 summarizes the different data modalities used in AI-based predictive models and their influence on predictive performance.

**Table 7:** Comparison of data modalities in AI-based prediction models.

#	Data type	Usage	Impact on performance	References
1	Clinical data	Widely used	Moderate predictive accuracy; limited representation of complex physiology	Zhang J. et al., 2025; Syngelaki et al., 2025
2	Biomarkers	Increasing use	Enhances early detection through biochemical indicators	Zhang L. et al., 2025
3	Electronic Health Records (EHR)	Large-scale datasets	Improves generalizability and population-level modeling	Artzi et al., 2020; Zhang, 2026
4	Continuous glucose monitoring (CGM) / Time-series data	Emerging	Captures temporal dynamics and improves prediction of disease progression	Liang, 2023; Sharifi-Heris, 2023
5	Multi-modal data	Integrated approaches	Achieves the highest predictive performance through complementary data fusion	Guo et al., 2025; Du et al., 2026

Table 7 shows that multimodal data models generally achieved superior predictive performance compared with single-source models, which underscores the significance of combining clinical, biochemical, and temporal data to enhance predictions. The most frequently reported predictive factors are maternal obesity, signs of insulin resistance (e.g., TyG index), blood pressure, and inflammatory [e.g., soluble fms-like tyrosine kinase-1 (sFlt-1)] and angiogenic (e.g., placental growth factor (PlGF)) biomarkers (Guo, 2024; Qiao et al., 2025). These characteristics indicate the common metabolic and vascular pathways of both conditions, supporting the biological feasibility of AI-based predictions.

Most studies employed internal validation techniques, such as cross-validation and split-sample validation, whereas comparatively few performed external validation (Liu et al., 2026). This limits the generalizability and clinical applicability of many published AI models. Furthermore, the lack of standardized validation frameworks and reporting practices hinders meaningful comparisons across studies. Table 8 summarizes the validation strategies used in AI-based predictive models.

**Table 8:** Validation strategies employed in AI-based predictive models for gestational diabetes mellitus and preeclampsia.

#	Validation strategy	Frequency	Strengths/limitations	Representative studies
1	Internal validation	Most common	Provides initial assessment of model performance but may overestimate predictive accuracy because training and testing are performed within the same population.	Zhang, 2026; Ji et al., 2025; Pinto et al., 2023; Deng et al., 2024
2	Cross-validation	Common	Reduces overfitting and improves robustness; however, performance remains dependent on the characteristics of the original dataset.	Maric et al., 2020; Du et al., 2022; Lu et al., 2021; Hu et al., 2023
3	External validation	Limited	Provides stronger evidence of model generalizability across independent populations but remains underutilized in AI prediction studies.	Arora et al., 2025; Eberhard et al., 2025; Zhang J. et al., 2025; Chan et al., 2023
4	Prospective validation	Rare	Represents the highest level of clinical validation by evaluating model performance in real-world settings; however, prospective studies are resource-intensive and relatively uncommon.	Wu Y. et al., 2025; Kurt et al., 2023

Table 8 illustrates the common emphasis on internal validation and limited use of external and prospective validation techniques, which critically limits the generalizability and clinical utility of the current AI models (Liu et al., 2026).

In recent years, the use of explainable artificial intelligence techniques for developing models has increased. The key predictors identified were maternal age, body mass index (BMI), glucose, and inflammation markers using feature importance analysis and SHAP-based explanations. Several studies have been conducted on the integration of predictive models into clinical decision support systems. These systems have shown promise for real-time risk assessment and early intervention but have not yet been adopted in routine clinical practice. The lack of a standardized framework for integrating AI models into healthcare systems demonstrates the vast gap between model development and clinical implementation.

Most AI models were developed to independently predict GDM and preeclampsia, which is important. Despite their shared pathophysiology, very few studies have investigated the joint prediction of these two conditions, representing a critical gap in the literature.

Overall, the evidence synthesized from Tables 2, 7, 8 demonstrates a clear transition from traditional single-source predictive models toward integrated multimodal and data-driven AI approaches (Chen Z., 2025; Zhang, 2025). These models show considerable promise for improving early risk stratification and supporting personalized maternal care through enhanced predictive performance. However, their successful translation into routine clinical practice remains constrained by limited external validation, insufficient model interpretability, the absence of standardized validation frameworks, and challenges associated with real-world clinical implementation.

The observed epidemiological association between gestational diabetes mellitus and preeclampsia provides a strong basis for constructing integrated AI-based predictive models. There are common metabolic and vascular risk factors for these diseases. The characteristics of these factors are similar to those used in machine learning models (Tang J., 2025; Qin, 2025). This similarity suggests that multimodal AI approaches based on biological knowledge could improve the predictive accuracy and clinical relevance of these models in the future.

This review identified several limitations in the studies included. Many studies were retrospective observational studies, which are subject to selection and residual confounding biases. Heterogeneity among studies was also attributed to variability in the diagnostic criteria for gestational diabetes mellitus and preeclampsia. Additionally, population demographics, sample size, and feature selection strategies could have contributed to the generalizability of the findings. The limited use of external validation and prospective study designs also limits the translation of predictive models into clinical practice (Eberhard et al., 2025, 2024). In addition, the good predictive performance of AI models is also strikingly in line with the known epidemiological and pathophysiological relationships between gestational diabetes mellitus and preeclampsia (Wu H. et al., 2025; Tang W., 2025). This suggests that AI-based models learn the underlying biological mechanisms and provide clinically relevant risk predictions.

### 4.4 Epidemiological association between GDM and preeclampsia

Gestational diabetes mellitus and preeclampsia have been described as being associated with each other in previous studies (Ye, 2025; Li, 2025). Women with GDM have an increased risk of preeclampsia of approximately 1.5-3-fold compared to normoglycemic pregnancies. This association was consistent across populations, study designs and clinical settings.

Common maternal risk factors, such as obesity, advanced maternal age, insulin resistance, and metabolic syndrome, have been repeatedly reported (Abdi, 2025; Tagami, 2026; Mensah, 2026), suggesting a common cardiometabolic background for both diseases.

### 4.5 Quantitative and consistency synthesis

Although the included studies differed in terms of population characteristics, study design, diagnostic criteria, and analytical approaches, the overall body of evidence consistently demonstrated a positive association between gestational diabetes mellitus (GDM) and preeclampsia. Across observational cohorts, retrospective studies, and biomarker investigations, women diagnosed with GDM were generally reported to have an increased likelihood of subsequently developing preeclampsia compared with normoglycaemic pregnancies (Li, 2025; Ye, 2025; Wu H. et al., 2025; Umer, 2025).

Beyond this epidemiological association, several studies identified common metabolic and cardiovascular risk factors underlying both conditions. Maternal obesity, dyslipidaemia, and adverse metabolic profiles were repeatedly associated with an increased risk of both GDM and preeclampsia, supporting the hypothesis that these disorders share overlapping pathophysiological pathways rather than representing entirely independent pregnancy complications (Abdi, 2025; Qin, 2025; Tang J., 2025). Furthermore, studies involving twin pregnancies demonstrated that the coexistence of GDM and hypertensive disorders was associated with poorer maternal and neonatal outcomes, reinforcing the clinical importance of early identification and risk stratification (Wu H. et al., 2025; Tang W., 2025).

Representative studies illustrating the association between GDM and preeclampsia are summarized in Table 9. Although the reported magnitude of risk varied across studies, the direction of the association remained remarkably consistent, irrespective of study design or geographic setting.

**Table 9:** Representative studies describing the association between gestational diabetes mellitus (GDM) and preeclampsia (PE).

Study	Study design	Principal findings
Li (2025)	Observational cohort study	Demonstrated a significant association between gestational diabetes mellitus and subsequent development of preeclampsia, supporting the coexistence of shared maternal risk factors.
Ye (2025)	Population-based cohort study	Confirmed gestational diabetes mellitus as an independent predictor of preeclampsia after adjustment for maternal demographic and clinical characteristics.
Wu H. et al. (2025)	Retrospective cohort study	Reported that twin pregnancies complicated by gestational diabetes had a significantly higher incidence of preeclampsia and adverse obstetric outcomes.
Tang W. (2025)	Cohort study	Showed that pregnancies affected by both gestational diabetes and hypertensive disorders experienced poorer maternal and neonatal outcomes than uncomplicated pregnancies.
Umer (2025)	Observational cohort study	Demonstrated increased maternal and neonatal morbidity when gestational diabetes coexisted with preeclampsia, highlighting the clinical importance of combined risk assessment.
Mustafa et al. (2025)	Population-based machine learning study	Large-scale machine learning analysis identified gestational diabetes and hypertensive disorders as strongly associated pregnancy complications with overlapping risk profiles and adverse outcomes.
Syngelaki et al. (2025)	Large prospective cohort study	Validated gestational diabetes mellitus as a significant predictor of adverse pregnancy outcomes, including hypertensive disorders, in a large clinical population.

The available evidence demonstrates a highly consistent relationship between GDM and preeclampsia across diverse study populations and clinical settings. Although differences in study design, participant characteristics, diagnostic criteria, and statistical adjustment contribute to variability in the reported magnitude of association, the convergence of findings across independent investigations provides robust support for a clinically meaningful relationship between these two pregnancy complications. These findings further justify the development of integrated risk prediction models that simultaneously evaluate both disorders and reinforce the importance of early screening strategies capable of identifying women at elevated obstetric risk.

### 4.6 Sources of heterogeneity

Variability in the reported effect sizes was attributed to differences in diagnostic criteria, population characteristics, study design, and adjustment for confounding variables (Liu et al., 2026). Such heterogeneity does not affect the direction of the association and supports the robustness of this association.

### 4.7 Shared risk factors and pathophysiology

Evidence synthesized from the included studies indicates that gestational diabetes mellitus (GDM) and preeclampsia share several interrelated pathophysiological mechanisms, including insulin resistance, systemic inflammation, endothelial dysfunction, oxidative stress, placental abnormalities, and metabolic dysregulation. These shared biological pathways provide a plausible explanation for the frequent coexistence of both disorders and support the development of integrated risk prediction strategies (Abdi, 2025; Sun et al., 2023; Qin, 2025; Tossetta, 2026; Furuta, 2026; Petkova-Parlapanska, 2025).

Table 10 summarizes the principal metabolic, inflammatory, and placental mechanisms that contribute to the development of both gestational diabetes mellitus and preeclampsia.

**Table 10:** Shared metabolic and pathophysiological risk factors linking gestational diabetes mellitus and preeclampsia.

Risk factor	Evidence strength	Description
Maternal obesity	Strong	A major metabolic risk factor associated with increased susceptibility to both gestational diabetes mellitus and preeclampsia through insulin resistance and chronic low-grade inflammation (Abdi, 2025).
Insulin resistance	Strong	Central metabolic mechanism contributing to impaired glucose regulation, endothelial dysfunction, and increased risk of hypertensive disorders during pregnancy (Sun et al., 2023).
Systemic inflammation	Strong	Elevated inflammatory mediators and immune dysregulation contribute to endothelial injury and progression of both gestational diabetes mellitus and preeclampsia (Sun et al., 2022; Ahmed, 2026).
Placental dysfunction	Strong	Abnormal placental development, impaired angiogenesis, and altered trophoblast function represent common pathological mechanisms underlying both disorders (Tossetta, 2026; Fiskaa, 2026; Furuta, 2026).
Metabolic dysregulation	Moderate–Strong	Altered lipid metabolism and cardiometabolic disturbances are consistently associated with increased risk of gestational diabetes mellitus and hypertensive pregnancy complications (Qin, 2025; Tang J., 2025).
Oxidative stress	Moderate	Oxidative stress contributes to placental dysfunction, endothelial injury, and progression of adverse pregnancy outcomes (Petkova-Parlapanska, 2025).

### 4.8 Maternal and neonatal outcomes

GDM and preeclampsia are associated with increased morbidity in mothers and children (Li, 2025; Umer, 2025). The maternal outcomes studied were hypertension, cesarean delivery, and chronic cardiac-metabolic conditions. The neonatal outcomes reported were preterm birth, macrosomia, hypoglycemia, and NICU admission (Wu and Chen, 2026; Tang W., 2025).

### 4.9 Emerging digital health and clinical integration

Advancements in technology, such as continuous glucose monitoring, wearable devices, and digital health platforms, have improved the ability to monitor and provide personalized care in real time (Liang, 2023). This has been followed by difficulty in implementing AI models in real-world clinical settings owing to issues of validation, interpretability, and integration with healthcare systems (Ahmed et al., 2025; Liu et al., 2026).

### 4.10 Summary of key findings

The findings showed that GDM is strongly and consistently correlated with preeclampsia, both of which have common metabolic and vascular pathways (Ye, 2025; Li, 2025). Multimodal data are effectively used with AI-based predictive models to outperform traditional approaches for early risk stratification (Zaky et al., 2025; Ji et al., 2025). Although promising, they have several weaknesses in terms of validation, interpretation, and clinical integration, and indicate the scope for future research (Ahmed et al., 2025; Liu et al., 2026).

These results reinforce the necessity of developing integrated multimodal clinically applicable predictive strategies, for which the envisioned AI-based framework is provided in Section 5.

## 5 Proposed AI framework and future directions

The evidence synthesized in this systematic review indicates that future AI-based prediction of gestational diabetes mellitus (GDM) and preeclampsia (PE) requires greater integration of multimodal data, biologically informed modeling, rigorous validation, explainable artificial intelligence (AI), and clinical implementation strategies. Building upon these findings, we propose an evidence-derived conceptual framework that synthesizes the principal themes identified across the reviewed literature into a unified research model.

Rather than representing a validated prediction model or clinical decision-support system, the proposed framework serves as an evidence-informed conceptual synthesis designed to guide future AI model development, prospective validation, and clinical translation.

### 5.1 Evidence-derived conceptual framework

**Figure 8:**
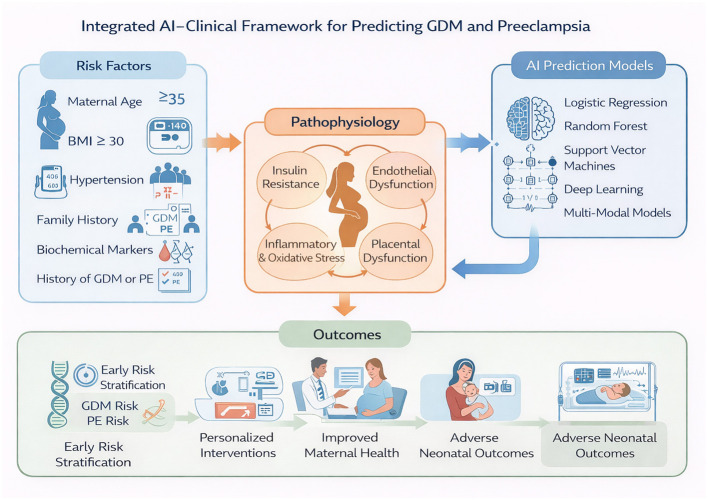
Evidence-derived conceptual framework for integrated AI-based prediction of gestational diabetes mellitus and preeclampsia. The framework synthesizes the principal findings of this systematic review by integrating established clinical risk factors, shared pathophysiological mechanisms, artificial intelligence methodologies, and anticipated clinical outcomes to guide future development of integrated AI prediction systems.

The quantitative evidence synthesized in this review suggests that prediction performance generally improves when multimodal data sources are integrated with complementary artificial intelligence (AI) methodologies. However, the existing literature remains characterized by considerable methodological heterogeneity, inconsistent validation strategies, and limited external validation, which restrict the generalizability and clinical translation of current AI models. Building upon these findings, Figure 8 presents an evidence-derived conceptual framework that synthesizes the principal themes identified across the reviewed literature, including multimodal data integration, shared pathophysiological mechanisms, AI prediction approaches, and clinical translation. Rather than representing a validated prediction model, the framework provides an evidence-informed roadmap for future development, implementation, and evaluation of integrated AI systems for predicting gestational diabetes mellitus (GDM) and preeclampsia (PE).

Unlike a clinical prediction model, the proposed framework is not intended to generate risk estimates or serve as a validated clinical decision-support system. Instead, it integrates the evidence obtained from the reviewed studies into a unified conceptual architecture linking established risk factors, shared pathophysiological mechanisms, AI methodologies, and anticipated clinical outcomes. The framework therefore represents a synthesis of the current evidence base and provides a roadmap for future development and evaluation of integrated AI prediction systems.

The framework comprises four complementary components.

**Risk Factors**. The first component represents the major clinical predictors consistently identified across the included studies, including maternal age, obesity, hypertension, family history, previous pregnancy complications, and biochemical biomarkers. These variables form the principal inputs for AI-based prediction models and reflect the established clinical determinants of GDM and PE reported throughout the reviewed literature.

**Shared Pathophysiological Mechanisms**. The second component highlights the biological pathways common to both GDM and PE, including insulin resistance, endothelial dysfunction, placental dysfunction, inflammation, and oxidative stress. Incorporating these mechanisms provides biological context for AI prediction and supports the development of models that extend beyond purely statistical associations toward greater clinical interpretability.

**Artificial Intelligence Prediction Models**. The third component synthesizes the AI methodologies identified in this review, including logistic regression, random forests, support vector machines, deep learning architectures, and multimodal AI models. The quantitative evidence synthesis demonstrated a clear progression from conventional machine learning approaches toward multimodal AI frameworks capable of integrating heterogeneous clinical, imaging, biochemical, and longitudinal data. Studies employing multimodal approaches generally demonstrated superior predictive performance, highlighting the potential advantages of integrating complementary sources of information.

**Clinical Translation**. The final component illustrates the intended pathway from AI prediction to clinical application. Accurate early prediction has the potential to facilitate risk stratification, individualized monitoring, targeted preventive interventions, and improved maternal and neonatal outcomes. These applications represent the long-term clinical objectives identified across the reviewed literature rather than outcomes demonstrated by the present review.

The framework integrates the principal evidence generated by this systematic review into a coherent conceptual model that links clinical risk factors, disease biology, AI methodologies, and clinical translation. In contrast to many existing studies that focus on individual prediction models or isolated data sources, the framework emphasizes the complementary relationships among multimodal data integration, biologically informed modeling, explainable AI, rigorous validation, and clinical implementation. Accordingly, it provides an evidence-informed foundation for future research aimed at developing integrated AI systems capable of jointly predicting gestational diabetes mellitus and preeclampsia.

A limitation of the proposed framework is that it represents a conceptual synthesis derived from the existing evidence rather than an implemented or empirically validated prediction model. Consequently, its clinical utility remains to be established through prospective multicentre implementation studies, external validation across diverse populations, and evaluation within real-world clinical decision-support systems before routine clinical adoption can be recommended.

### 5.2 Future research directions

The evidence synthesized in this systematic review identified several important methodological and translational gaps that should guide future research on AI-based prediction of gestational diabetes mellitus (GDM) and preeclampsia (PE). Addressing these limitations will be essential for improving the robustness, generalizability, interpretability, and clinical implementation of future AI prediction systems.

- **Integrated Prediction of GDM and Preeclampsia**. The quantitative evidence synthesis demonstrated that only a limited number of studies developed integrated prediction models for both GDM and PE or related hypertensive disorders of pregnancy. Future research should therefore prioritize unified AI frameworks capable of simultaneously predicting multiple pregnancy complications by leveraging their shared metabolic, inflammatory, vascular, and placental mechanisms rather than developing separate disease-specific models (Ahmed et al., 2025).
- **Standardization of Methodological Practices**. Considerable heterogeneity was observed across datasets, predictor selection, feature engineering, validation strategies, and reporting practices. Future studies should adopt standardized methodological and reporting frameworks to improve reproducibility, facilitate comparison across studies, and strengthen the evidence base for clinical translation (Liu et al., 2026).
- **Expansion of Multimodal Data Integration**. The review demonstrated that multimodal AI approaches generally achieved superior predictive performance compared with single-modality models. Future studies should further investigate the integration of clinical data, electronic health records, imaging, biomarkers, continuous glucose monitoring, wearable sensors, and multi-omics data to improve prediction accuracy while providing greater biological insight into disease mechanisms (Ji et al., 2025; Zaky et al., 2025).
- **Explainable and Trustworthy Artificial Intelligence**. Improving predictive performance alone is insufficient for clinical adoption. Future research should place greater emphasis on explainable AI techniques, including SHAP, LIME, and other interpretable approaches, to improve transparency, clinician confidence, regulatory acceptance, and patient trust while maintaining predictive accuracy (Patel, 2023; Chen J., 2025).
- **Robust External Validation and Clinical Implementation**. One of the principal methodological limitations identified in this review was the limited use of external and prospective validation. Future AI models should therefore undergo multicentre external validation across diverse populations followed by prospective implementation studies to establish their robustness, generalizability, and real-world clinical utility before routine deployment within healthcare systems (Popova, 2023; Liu et al., 2026).
- **Toward Precision Maternal Healthcare**. Ultimately, future AI systems should progress beyond isolated prediction tasks toward integrated clinical decision-support systems capable of supporting personalized risk assessment, early intervention, and individualized management strategies for high-risk pregnancies. Such developments have the potential to advance precision medicine while improving maternal and neonatal outcomes.

Overall, the findings of this systematic review indicate that future research should move beyond isolated prediction models toward integrated, multimodal, explainable, and clinically validated AI systems. Achieving this objective will require standardized methodologies, rigorous external validation, prospective clinical evaluation, and closer integration between artificial intelligence, clinical expertise, and biological understanding to support safe and effective implementation in maternal healthcare.

### 5.3 Implications for clinical practice

The evidence synthesized in this systematic review suggests that artificial intelligence (AI) has considerable potential to complement existing clinical assessment for the early identification of women at risk of gestational diabetes mellitus (GDM) and preeclampsia (PE). In particular, the reviewed studies demonstrated that multimodal AI approaches integrating heterogeneous clinical, biochemical, imaging, and longitudinal data generally achieved superior predictive performance compared with single-modality models. These findings indicate that future AI systems may enhance risk stratification by combining diverse sources of patient information within a unified predictive framework (Zaky et al., 2025; Ji et al., 2025; Li, 2025).

The evidence-derived conceptual framework proposed in this review provides a structured roadmap for the future development of clinically relevant AI prediction systems by integrating established risk factors, shared pathophysiological mechanisms, multimodal data, explainable AI, rigorous validation strategies, and clinical decision-support considerations. If successfully implemented and prospectively validated, such integrated approaches could support earlier risk identification, individualized monitoring, targeted preventive interventions, and more personalized maternal healthcare.

Nevertheless, the current evidence also highlights important barriers to clinical implementation. Considerable methodological heterogeneity, limited external validation, inconsistent reporting practices, and the relatively small number of studies investigating integrated prediction of both GDM and PE continue to restrict the clinical translation of existing AI models. Addressing these limitations through standardized methodologies, multicentre external validation, prospective evaluation, and explainable AI will be essential before routine implementation in healthcare settings can be recommended.

Accordingly, the proposed framework should be interpreted as an evidence-derived conceptual guide for future research and system development rather than as a validated clinical prediction model. Its principal contribution is to synthesize the current evidence into a coherent framework capable of guiding the design, implementation, and evaluation of future integrated, interpretable, and clinically deployable AI systems for precision maternal healthcare.

## 6 Discussion

This systematic review synthesizes epidemiological, clinical, mechanistic, and artificial intelligence (AI)-based evidence to provide an integrated understanding of the relationship between gestational diabetes mellitus (GDM) and preeclampsia (Wu H. et al., 2025). The synthesized evidence indicates that these conditions should not be viewed as independent pregnancy complications but rather as interconnected manifestations of shared cardiometabolic and vascular dysfunction (Ye, 2025; Li, 2025). By integrating conventional clinical evidence with emerging AI-based predictive research, this review provides an evidence-informed perspective that may inform future research, predictive model development, and the translation of AI into maternal healthcare.

One of the common themes emerging from the reviewed literature is the presence of shared maternal risk factors, particularly obesity, advanced maternal age, insulin resistance, and metabolic syndrome (Abdi, 2025; Tagami, 2026; Mensah, 2026). These factors are well-established indicators of underlying cardiometabolic vulnerability that often precedes pregnancy and increases the risk of developing GDM and preeclampsia. Rather than acting independently, these risk factors interact to amplify metabolic and vascular stress during pregnancy, supporting the concept of pregnancy as a physiological stress test that can reveal underlying cardiometabolic dysfunction.

The association between GDM and preeclampsia is, therefore, biologically plausible. Shared mechanisms have been identified in experimental animal models, including insulin resistance, systemic inflammation, endothelial dysfunction, oxidative stress, and placental abnormalities (Petkova-Parlapanska, 2025; Furuta, 2026). All these processes are interconnected and form a complex pathological network, in which metabolic abnormalities contribute to vascular damage and placental insufficiency, which in turn worsen during pregnancy, leading to the onset of hypertensive disorders. Importantly, these mechanistic findings align closely with the important predictive features observed in AI-based models, such as those related to insulin resistance and inflammation (Guo, 2024; Sun et al., 2023). AI-based predictors and biological evidence further strengthen the biological validity and clinical relevance of the new predictive algorithms.

GDM and preeclampsia are both associated with substantial maternal and neonatal morbidity, contributing to adverse pregnancy outcomes and increased healthcare burden (Umer, 2025; Wu H. et al., 2025). These conditions significantly increase women's risk of developing long-term cardiometabolic diseases, such as type 2 diabetes and heart disease, and place their children at risk of poor metabolic outcomes later in life. This result highlights the significance of early detection and targeted interventions, not only for pregnancy outcomes but also for the health of future generations.

One of the principal contributions of this review is the synthesis of evidence on AI-based predictive models within a broader epidemiological, clinical, and mechanistic context, providing an integrated perspective on the early prediction of gestational diabetes mellitus (GDM) and preeclampsia. Across the reviewed studies, AI-based models consistently demonstrated promising predictive performance, particularly when multimodal data were integrated to predict disease risk (Tiruneh et al., 2024; Sharifi-Heris, 2023; Zhang, 2025). These models are capable of capturing complex nonlinear relationships among clinical variables, biochemical markers, and longitudinal health data that are often difficult to model using conventional statistical approaches. The findings are consistent with the broader body of evidence suggesting that machine learning approaches can improve obstetric risk prediction by leveraging heterogeneous and high-dimensional datasets. However, unlike previous reviews that primarily summarize AI model performance, this study integrates epidemiological, mechanistic, and AI-based evidence within a unified conceptual framework that provides an evidence-informed foundation for future predictive model development, prospective validation, and clinical translation.

Notably, many of the predictive variables identified by AI models, including indices of insulin resistance, angiogenic biomarkers, inflammatory markers, and maternal clinical characteristics, correspond closely to the established pathophysiological mechanisms underlying GDM and preeclampsia (Guo, 2024; Li, 2026). This convergence suggests that AI-based prediction extends beyond statistical pattern recognition by capturing clinically meaningful biological signals associated with disease development. The alignment between AI-derived predictive features and established biological mechanisms enhances the biological plausibility and interpretability of these models, providing a stronger scientific rationale for their future development, prospective validation, and evaluation in clinical settings.

Despite these developments, there are several obstacles to the clinical use of AI models. Most studies rely primarily on internal validation, with relatively few incorporating external validation across diverse populations. Consequently, the generalizability of these models to different clinical settings and patient populations remains uncertain. Furthermore, inconsistencies in feature selection, reporting practices, and dataset characteristics hinder direct comparison between models and contribute to substantial methodological heterogeneity. In addition, the limited adoption of explainable AI (XAI) methods reduces model transparency, potentially limiting clinician trust and hindering clinical implementation (Patel, 2023; Chen J., 2025). Moreover, most existing AI models predict GDM and preeclampsia independently despite their shared pathophysiological mechanisms. This highlights an important gap in current prediction research and provides further justification for developing integrated predictive approaches.

The synthesized evidence supports moving beyond the traditional view of GDM and preeclampsia as distinct pregnancy complications toward a shared cardiometabolic paradigm. This paradigm is characterized by shared maternal risk factors that contribute to a cascade of metabolic, vascular, and placental dysfunction, giving rise to distinct yet overlapping clinical phenotypes. Within this context, AI-based models provide a complementary analytical approach capable of integrating complex clinical, biological, and temporal information into individualized risk prediction frameworks (Chen Z., 2025). Their ability to capture nonlinear interactions across diverse data sources highlights their potential to support future precision risk assessment, although prospective validation remains necessary before routine clinical application.

These findings provide the theoretical rationale for proposing an integrated conceptual framework that integrates the key evidence synthesized in this systematic review. While the framework illustrates how multimodal data, biological mechanisms, explainable AI, and standardized validation may be combined within a unified architecture, it has not been empirically evaluated. Its primary contribution therefore lies in providing an evidence-informed foundation for future implementation, prospective validation, and clinical evaluation rather than demonstrating immediate clinical applicability.

### 6.1 Critical interpretation and remaining uncertainties

Although the association between gestational diabetes mellitus (GDM) and preeclampsia is well established, several important uncertainties remain regarding the underlying mechanisms and causal pathways. Variations in reported effect sizes across studies likely reflect differences in population characteristics, diagnostic criteria, study design, and methodological approaches (Özcan and Peker, 2025). Furthermore, the direction and nature of the causal relationship remain uncertain. Although metabolic dysfunction associated with GDM may contribute to hypertensive complications during pregnancy, preeclampsia is a heterogeneous disorder in which placental dysfunction, vascular abnormalities, and maternal susceptibility may independently influence disease onset.

Mechanistic uncertainty also persists. Although several shared biological pathways have been identified, the temporal sequence and relative contribution of metabolic, vascular, inflammatory, and placental mechanisms remain incompletely understood (Petkova-Parlapanska, 2025; Furuta, 2026). Future longitudinal mechanistic studies integrating clinical, molecular, and AI-derived data are therefore needed to clarify disease progression, establish temporal relationships among these pathways, and support the development of biologically informed predictive models.

### 6.2 Causality and integrated pathophysiological interpretation

The relationship between gestational diabetes mellitus (GDM) and preeclampsia appears to be more complex than a simple bidirectional association. Instead, the available evidence suggests that these conditions represent interconnected manifestations of shared cardiometabolic and placental dysfunction arising from overlapping pathophysiological pathways (Ahmed et al., 2025). Temporal evidence suggests that metabolic disturbances may predispose susceptible women to subsequent vascular complications, whereas placental dysfunction may independently contribute to the development of early-onset preeclampsia.

Emerging genetic, epigenetic, and molecular evidence further supports the hypothesis of shared biological susceptibility between GDM and preeclampsia. However, important confounding factors, including obesity, maternal age, and pre-existing metabolic disorders, complicate causal inference and make it difficult to distinguish shared susceptibility from direct causal relationships (Abdi, 2025; Mensah, 2026). Overall, the synthesized evidence more strongly supports a model of shared pathophysiology than a simple linear cause-and-effect relationship between GDM and preeclampsia.

### 6.3 Clinical and translational implications

The synthesis of clinical, biological, and AI-based evidence provides an important foundation for improving risk assessment and clinical decision-making in maternal healthcare. The findings support consideration of bidirectional screening strategies, whereby women diagnosed with gestational diabetes mellitus (GDM) are monitored for subsequent hypertensive complications and women with preeclampsia are assessed for metabolic risk. Such an approach may facilitate earlier risk identification and more targeted clinical interventions (Tang W., 2025).

AI-based predictive models also have the potential to support clinical practice by improving patient risk stratification and facilitating more individualized management strategies. If prospectively validated and successfully integrated into clinical workflows, these models could be incorporated into clinical decision support systems (CDSS) to assist with early risk prediction, ongoing risk monitoring, and personalized clinical decision-making (Du et al., 2026).

Overall, the evidence synthesized in this review highlights the importance of integrated screening strategies and supports the future development of AI-assisted clinical decision-support tools to enable more timely and individualized maternal care.

### 6.4 Public health implications

The rising global burden of gestational diabetes mellitus (GDM) and preeclampsia underscores the need for integrated preventive strategies to improve maternal cardiometabolic health from a public health perspective (Barrell, 2025). Early screening programmes, lifestyle interventions, and digital health technologies have the potential to reduce disease burden, improve maternal and neonatal outcomes, and facilitate earlier risk stratification during pregnancy.

The convergence of epidemiological, mechanistic, and AI-based evidence synthesized in this review further supports the need for integrated risk assessment strategies capable of informing preventive, personalized, and population-level maternal healthcare. Future research should focus on validating integrated AI-supported approaches across diverse healthcare settings to strengthen their contribution to public health policy and maternal care.

### 6.5 Limitations and strengths

Some limitations of this review include the observational nature of the included studies, variations in study design, clinical definitions, and AI methodologies, as well as the potential for publication bias. In addition, the heterogeneity in model development, feature selection, validation strategies, and reporting limited direct quantitative comparison across studies. A further limitation is that the proposed AI framework represents an evidence-informed conceptual synthesis and was neither implemented nor empirically validated as part of this review. Consequently, its predictive performance, clinical utility, and generalizability remain to be established through future implementation and prospective validation. Nevertheless, the strengths of this review include its comprehensive synthesis of recent evidence, adherence to PRISMA guidelines, and integration of epidemiological, clinical, mechanistic, and AI-based evidence into a unified conceptual framework.

By integrating epidemiological, clinical, mechanistic, and AI-based evidence, this review provides a comprehensive and multidimensional perspective on the relationship between GDM and preeclampsia. The synthesized evidence supports the view that these conditions share common cardiometabolic and pathophysiological pathways (Nguyen, 2024). Furthermore, the integration of AI-based prediction with established biological and clinical knowledge provides an evidence-informed foundation for future research aimed at developing more interpretable, robust, and clinically relevant predictive models (Sharifi-Heris, 2023; Zhang, 2025). However, translating these advances into routine clinical practice will require rigorous implementation, external validation, improved interpretability, and seamless integration into existing healthcare systems (Özcan and Peker, 2025).

Ultimately, the development of integrated, interpretable, and clinically validated AI systems will be essential for supporting the transition from reactive pregnancy management toward more proactive and precision-oriented maternal healthcare.

## 7 Conclusion

This systematic review synthesized the current evidence on artificial intelligence (AI)-based prediction of gestational diabetes mellitus (GDM) and preeclampsia (PE), integrating epidemiological, clinical, mechanistic, and computational perspectives. The findings indicate that these pregnancy complications share important cardiometabolic, vascular, inflammatory, and placental mechanisms that provide a strong rationale for developing integrated AI-based prediction strategies rather than isolated disease-specific models. The review further demonstrates that multimodal data integration, rigorous validation strategies, and explainable artificial intelligence are key methodological components for improving predictive performance and facilitating future clinical translation.

An important contribution of this review is the development of an evidence-derived conceptual framework that synthesizes the principal findings of the reviewed literature into a unified architecture linking established clinical risk factors, shared pathophysiological mechanisms, AI methodologies, and potential clinical applications. Rather than representing a validated prediction model, the framework provides an evidence-informed roadmap to guide future research, implementation, and evaluation of integrated AI systems for predicting GDM and PE.

Despite the encouraging progress reported across the reviewed studies, several important challenges remain. These include considerable methodological heterogeneity, limited external validation, insufficient explainability, fragmented prediction of individual pregnancy complications, and limited integration of AI models into routine clinical workflows. Addressing these challenges will require standardized methodological practices, large multicentre longitudinal datasets, prospective validation, and rigorous evaluation within diverse real-world healthcare settings.

Overall, this review provides a comprehensive synthesis of the current evidence and identifies key priorities for advancing AI-assisted prediction of pregnancy complications. Future research should focus on developing integrated, multimodal, interpretable, and clinically validated AI systems capable of supporting earlier risk identification, personalized maternal care, and improved maternal and neonatal outcomes.
