# Supplementary material for: Artificial intelligence for early prediction of gestational diabetes mellitus and preeclampsia: a systematic review of machine learning models and clinical decision support systems
Source: Front Artif Intell. 2026 Jul 30;9:1890320. doi: 10.3389/frai.2026.1890320 (PMC13469252; doi:10.3389/frai.2026.1890320)
Supplement: Supplementary file 1 [file Table_1.pdf]

Supplementary Material

The supplementary material (Supplementary Table 1) associated with this article provides a structured classification of the studies included in the overall evidence synthesis. To improve methodological transparency, the studies were classified according to their primary contribution to the review, distinguishing primary AI prediction studies from AI-supporting, clinical/epidemiological, and mechanistic/biomarker evidence.

Table 1: Supplementary Table S1. Evidence classification of the 120 studies included in the overall evidence synthesis (2020–2026).

Ref	Author (Reference)	Study Classification	Evidence Category	Primary Contribution to the Review	Quantitative AI Analysis
1	Guo et al. Guo et al. [2025]	Multimodal Artificial Intelligence	Category I – Primary AI Prediction Study	Development and validation of a multimodal AI model for prediction of gestational diabetes mellitus and hypertensive disorders of pregnancy	Yes
2	Du et al. Du et al. [2026]	Deep Learning	Category I – Primary AI Prediction Study	Development of a multimodal deep learning fusion model for early prediction of gestational diabetes mellitus and preeclampsia	Yes
3	Hassan et al. Hassan et al. [2025]	Explainable Machine Learning	Category I – Primary AI Prediction Study	Development of an explainable ensemble learning model for gestational diabetes mellitus prediction	Yes
4	Cardenas-Urrelo et al. Cardenas-Urrelo et al. [2026]	Machine Learning	Category I – Primary AI Prediction Study	Clinical, haemodynamic and biochemical machine learning model for prediction of preeclampsia	Yes
5	Sun et al. Sun et al. [2022]	Multimodal Deep Learning	Category I – Primary AI Prediction Study	Ultrasound image fusion using deep learning for gestational diabetes mellitus prediction	Yes
6	Sun et al. Sun et al. [2023]	Multimodal Deep Learning	Category I – Primary AI Prediction Study	Ultrasound image fusion model for prediction of hypertensive disorders of pregnancy	Yes

Table 1 (continued)

Ref	Author (Reference)	Study Classification	Evidence Category	Primary Contribution to the Review	Quantitative AI Analysis
7	Arora et al. Arora et al. [2025]	Deep Learning	Category I – Primary AI Prediction Study	Placental texture analysis integrated with deep learning for prediction of hypertensive disorders of pregnancy	Yes
8	Liu et al. Li et al. [2026]	Multimodal Artificial Intelligence	Category I – Primary AI Prediction Study	MRI-based multimodal artificial intelligence model for fetal brain disorder prediction	Yes
9	Dong et al. Dong et al. [2026]	Multimodal Artificial Intelligence	Category I – Primary AI Prediction Study	Early prediction of preeclampsia using a multimodal deep learning model integrating fundus photography and longitudinal clinical time-series data	Yes
10	Liao et al. Liao et al. [2022]	Multimodal Imaging Artificial Intelligence	Category II – AI Supporting Study	Advanced multimodal imaging framework supporting artificial intelligence-assisted placental assessment	No
11	Butler et al. Butler et al. [2024a]	Deep Learning	Category I – Primary AI Prediction Study	Development and external validation of a one-dimensional ResNet convolutional neural network for prediction of preeclampsia	AI Performance Synthesis
12	Wang et al. Wang et al. [2024]	Deep Learning	Category I – Primary AI Prediction Study	Self-attention convolutional neural network for gestational diabetes mellitus prediction	AI Performance Synthesis
13	Zhou et al. Zhou et al. [2024a]	Deep Learning Radiomics	Category I – Primary AI Prediction Study	ResNet50 radiomics model for gestational diabetes mellitus prediction with multicentre validation	AI Performance Synthesis
14	Eberhard et al. Eberhard et al. [2024]	Deep Learning Survival Analysis	Category I – Primary AI Prediction Study	DeepHit survival network for longitudinal prediction of preeclampsia risk	AI Performance Synthesis
15	Narayanan et al. Narayanan et al. [2026]	Explainable Machine Learning	Category I – Primary AI Prediction Study	Externally validated explainable TabNet model for gestational diabetes mellitus prediction	AI Performance Synthesis

Table 1 (continued)

Ref	Author (Reference)	Study Classification	Evidence Category	Primary Contribution to the Review	Quantitative AI Analysis
16	Wu et al. Wu et al. [2025a]	Deep Learning Imaging	Category I – Primary AI Prediction Study	Retinal deep learning model for early prediction of preeclampsia	AI Performance Synthesis
17	Zheng et al. Zheng [2022]	Deep Learning Radiomics	Category I – Primary AI Prediction Study	MRI radiomics-based deep learning model for prediction of preeclampsia and fetal growth restriction	AI Performance Synthesis
18	Zhou et al. Zhou et al. [2024b]	Deep Learning	Category I – Primary AI Prediction Study	Inception-ResNet-v2 convolutional neural network for prediction of preeclampsia	AI Performance Synthesis
19	Kurt et al. Kurt et al. [2023]	Deep Learning	Category I – Primary AI Prediction Study	Deep neural network with Bayesian optimisation for gestational diabetes mellitus prediction	AI Performance Synthesis
20	Bennett et al. Bennett et al. [2022]	Deep Learning	Category I – Primary AI Prediction Study	Cost-sensitive deep neural network for prediction of preeclampsia	AI Performance Synthesis
21	Eberhard et al. Eberhard et al. [2025]	Deep Learning	Category I – Primary AI Prediction Study	Development of a longitudinal deep learning model using maternal clinical data for prediction of preeclampsia	AI Performance Synthesis
22	Liang et al. Liang et al. [2025a]	Deep Learning	Category I – Primary AI Prediction Study	Deep neural network for early prediction of preeclampsia during pregnancy	AI Performance Synthesis
23	Artzi et al. Segal [2020]	Machine Learning	Category I – Primary AI Prediction Study	Electronic health record-based gradient boosting model for early prediction of gestational diabetes mellitus	AI Performance Synthesis
24	Marić et al. Winn [2020]	Machine Learning	Category I – Primary AI Prediction Study	Gradient boosting machine model for prediction of preeclampsia	AI Performance Synthesis
25	Zhang et al. Zhang et al. [2025a]	Machine Learning	Category I – Primary AI Prediction Study	LASSO-based machine learning model for prediction of gestational diabetes mellitus	AI Performance Synthesis
26	Ji et al. Wang [2025a]	Machine Learning	Category I – Primary AI Prediction Study	Multilayer perceptron model for gestational diabetes mellitus prediction	AI Performance Synthesis

Table 1 (continued)

Ref	Author (Reference)	Study Classification	Evidence Category	Primary Contribution to the Review	Quantitative AI Analysis
27	Zhang et al. Zhang [2022]	Machine Learning	Category I – Primary AI Prediction Study	Random forest model for prediction of gestational diabetes mellitus	AI Performance Synthesis
28	Pinto et al. Pinto et al. [2023]	Machine Learning	Category I – Primary AI Prediction Study	Integrated machine learning framework for gestational diabetes mellitus risk prediction	AI Performance Synthesis
29	Du et al. Wang [2022]	Machine Learning	Category I – Primary AI Prediction Study	Machine learning prediction model using maternal clinical characteristics for gestational diabetes mellitus	AI Performance Synthesis
30	Ye et al. Xiao [2020]	Machine Learning	Category I – Primary AI Prediction Study	Gradient boosting decision tree model for early prediction of gestational diabetes mellitus	AI Performance Synthesis
31	Ranjbar et al. Ranjbar et al. [2024]	Systematic Review of Artificial Intelligence	Category II – AI Supporting & Translational Study	Comprehensive synthesis of artificial intelligence methods for prediction of gestational diabetes mellitus and preeclampsia	AI Supporting Evidence
32	Ahmed et al. Ahmed et al. [2025]	Systematic Review of Artificial Intelligence	Category II – AI Supporting & Translational Study	Critical review of artificial intelligence models for early prediction of gestational diabetes mellitus and preeclampsia	AI Supporting Evidence
33	Liu et al. Liu et al. [2026]	Systematic Review and Meta-analysis	Category II – AI Supporting & Translational Study	Quantitative synthesis of machine learning models for pregnancy complications	AI Supporting Evidence
34	Aljameel et al. Aljameel et al. [2023]	Artificial Intelligence Review	Category II – AI Supporting & Translational Study	Review of machine learning approaches for pregnancy-related disease prediction	AI Supporting Evidence
35	Özcan and Peker Özcan and Peker [2025]	Artificial Intelligence Review	Category II – AI Supporting & Translational Study	Review of artificial intelligence applications in maternal healthcare	AI Supporting Evidence
36	Kokori et al. Kokori et al. [2024]	Artificial Intelligence Review	Category II – AI Supporting & Translational Study	Systematic review of AI applications in obstetric prediction models	AI Supporting Evidence

Table 1 (continued)

Ref	Author (Reference)	Study Classification	Evidence Category	Primary Contribution to the Review	Quantitative AI Analysis
37	Popova et al. Popova [2023]	Clinical Decision Support Systems	Category II – AI Supporting & Translational Study	Artificial intelligence-enabled clinical decision support for obstetric care	AI Supporting Evidence
38	Du et al. Wang [2022]	Clinical Decision Support Systems	Category II – AI Supporting & Translational Study	Development of AI-assisted clinical decision support systems for pregnancy management	AI Supporting Evidence
39	Patel et al. Patel [2023]	Explainable Artificial Intelligence	Category II – AI Supporting & Translational Study	Application of explainable AI techniques to improve interpretability of prediction models	AI Supporting Evidence
40	Chen et al. Chen [2025a]	Explainable Artificial Intelligence	Category II – AI Supporting & Translational Study	Explainable deep learning framework for clinically interpretable prediction models	AI Supporting Evidence
31	Ranjbar et al. Ranjbar et al. [2024]	Systematic Review of Artificial Intelligence	Category II – AI Supporting & Translational Study	Comprehensive synthesis of artificial intelligence methods for prediction of gestational diabetes mellitus and preeclampsia	AI Supporting Evidence
32	Ahmed et al. Ahmed et al. [2025]	Systematic Review of Artificial Intelligence	Category II – AI Supporting & Translational Study	Critical review of artificial intelligence models for early prediction of gestational diabetes mellitus and preeclampsia	AI Supporting Evidence
33	Liu et al. Liu et al. [2026]	Systematic Review and Meta-analysis	Category II – AI Supporting & Translational Study	Quantitative synthesis of machine learning models for pregnancy complications	AI Supporting Evidence
34	Aljameel et al. Aljameel et al. [2023]	Artificial Intelligence Review	Category II – AI Supporting & Translational Study	Review of machine learning approaches for pregnancy-related disease prediction	AI Supporting Evidence
35	Özcan and Peker Özcan and Peker [2025]	Artificial Intelligence Review	Category II – AI Supporting & Translational Study	Review of artificial intelligence applications in maternal healthcare	AI Supporting Evidence
36	Kokori et al. Kokori et al. [2024]	Artificial Intelligence Review	Category II – AI Supporting & Translational Study	Systematic review of AI applications in obstetric prediction models	AI Supporting Evidence

Table 1 (continued)

Ref	Author (Reference)	Study Classification	Evidence Category	Primary Contribution to the Review	Quantitative AI Analysis
37	Popova et al. Popova [2023]	Clinical Decision Support Systems	Category II – AI Supporting & Translational Study	Artificial intelligence-enabled clinical decision support for obstetric care	AI Supporting Evidence
38	Du et al. Du et al. [2026]	Clinical Decision Support Systems	Category II – AI Supporting & Translational Study	Development of AI-assisted clinical decision support systems for pregnancy management	AI Supporting Evidence
39	Patel et al. Patel [2023]	Explainable Artificial Intelligence	Category II – AI Supporting & Translational Study	Application of explainable AI techniques to improve interpretability of prediction models	AI Supporting Evidence
40	Chen et al. Chen [2025a]	Explainable Artificial Intelligence	Category II – AI Supporting & Translational Study	Explainable deep learning framework for clinically interpretable prediction models	AI Supporting Evidence
41	Scott et al. Metz [2021]	Machine Learning (Metabolomics)	Category I – Primary AI Prediction Study	Metabolomics-based machine learning approach for early prediction of gestational diabetes mellitus	AI Performance Synthesis
42	Hou et al. Nicolaides [2022]	Machine Learning	Category I – Primary AI Prediction Study	Random forest model for early prediction of gestational diabetes mellitus	AI Performance Synthesis
43	Mustafa et al. Mustafa et al. [2025]	Automated Machine Learning (AutoML)	Category I – Primary AI Prediction Study	AutoML framework for predicting gestational diabetes mellitus and hypertensive disorders of pregnancy	AI Performance Synthesis
44	Di Filippo et al. Welsh [2022]	Machine Learning	Category I – Primary AI Prediction Study	Machine learning model using maternal clinical variables for gestational diabetes mellitus prediction	AI Performance Synthesis
45	Kaya et al. Kaya et al. [2024a]	Machine Learning	Category I – Primary AI Prediction Study	XGBoost algorithm for gestational diabetes mellitus prediction	AI Performance Synthesis
46	Jader and Aminifar Aminifar [2022]	Ensemble Machine Learning	Category I – Primary AI Prediction Study	Ensemble learning model for gestational diabetes mellitus prediction	AI Performance Synthesis
47	Chan et al. Leung [2023]	Ensemble Machine Learning	Category I – Primary AI Prediction Study	Externally validated ensemble machine learning model for gestational diabetes mellitus prediction	AI Performance Synthesis

Table 1 (continued)

Ref	Author (Reference)	Study Classification	Evidence Category	Primary Contribution to the Review	Quantitative AI Analysis
48	Ahmed et al. Ahmed et al. [2025]	Artificial Intelligence Systematic Review	Category II – AI Supporting & Translational Study	Comprehensive synthesis of artificial intelligence applications for early detection of gestational diabetes mellitus and preeclampsia	AI Supporting Evidence
49	Zhang et al. Zhang [2023]	Machine Learning	Category II – AI Supporting & Translational Study	Overview of machine learning approaches for early prediction of gestational diabetes mellitus	AI Supporting Evidence
50	Sharifi-Heris et al. Sharifi-Heris [2023]	Artificial Intelligence and Wearable Sensors	Category II – AI Supporting & Translational Study	Integration of wearable sensor technologies with artificial intelligence for pregnancy monitoring	AI Supporting Evidence
51	Popova et al. Popova [2023]	Clinical Decision Support Systems	Category II – AI Supporting & Translational Study	Artificial intelligence-enabled clinical decision support systems for maternal healthcare	AI Supporting Evidence
52	Patel et al. Patel [2023]	Explainable Artificial Intelligence	Category II – AI Supporting & Translational Study	Explainable artificial intelligence for clinical prediction and decision support	AI Supporting Evidence
53	Liang et al. Liang [2023]	Clinical Artificial Intelligence	Category II – AI Supporting & Translational Study	Integration of continuous glucose monitoring with artificial intelligence for gestational diabetes management	AI Supporting Evidence
54	Zheng et al. Zheng [2022]	Machine Learning	Category I – Primary AI Prediction Study	Artificial intelligence models for prediction of preeclampsia	AI Performance Synthesis
55	Huang et al. Huang [2022]	Deep Learning	Category I – Primary AI Prediction Study	Artificial neural network models for pregnancy complication prediction	AI Performance Synthesis
56	Li et al. Li et al. [2024]	Machine Learning	Category I – Primary AI Prediction Study	Machine learning models for early prediction of preeclampsia	AI Performance Synthesis
57	Ballard et al. Ballard et al. [2024]	Machine Learning (Electronic Health Records)	Category I – Primary AI Prediction Study	Electronic health record-based machine learning model for pregnancy risk prediction	AI Performance Synthesis

Table 1 (continued)

Ref	Author (Reference)	Study Classification	Evidence Category	Primary Contribution to the Review	Quantitative AI Analysis
58	Tiruneh et al. Tiruneh et al. [2024]	Machine Learning Systematic Review	Category II – AI Supporting & Translational Study	Systematic review of machine learning applications for prediction of pregnancy complications	AI Supporting Evidence
59	Zhou et al. Zhou et al. [2024b]	Deep Learning	Category I – Primary AI Prediction Study	Retinal image-based deep learning model for prediction of hypertensive disorders of pregnancy	AI Performance Synthesis
60	Yu et al. Yu et al. [2024]	Artificial Intelligence	Category I – Primary AI Prediction Study	Cell-free DNA-based artificial intelligence model for early prediction of pregnancy complications	AI Performance Synthesis
61	Kovacheva et al. Kovacheva et al. [2024]	Machine Learning	Category I – Primary AI Prediction Study	Machine learning integrated with polygenic risk scores for prediction of preeclampsia	AI Performance Synthesis
62	Butler et al. Butler et al. [2024b]	Machine Learning	Category I – Primary AI Prediction Study	Artificial intelligence using electrocardiogram features for prediction of preeclampsia	AI Performance Synthesis
63	Kaya et al. Kaya et al. [2024b]	Machine Learning	Category I – Primary AI Prediction Study	Machine learning model for preconception risk assessment of preeclampsia	AI Performance Synthesis
64	Wu et al. Wu et al. [2025b]	Deep Learning	Category I – Primary AI Prediction Study	Artificial intelligence using retinal vascular imaging for early prediction of preeclampsia	AI Performance Synthesis
65	Adil et al. Adil et al. [2025]	Machine Learning	Category I – Primary AI Prediction Study	Cell-free DNA-based machine learning model for preeclampsia risk prediction	AI Performance Synthesis
66	Zaky et al. Zaky et al. [2025]	Machine Learning Prediction Model	Category I – Primary AI Prediction Study	First-trimester machine learning model for early prediction of gestational diabetes mellitus using clinical biomarkers	AI Performance Synthesis
67	Chen et al. Chen [2025a]	Explainable Artificial Intelligence	Category II – AI Supporting & Translational Study	Explainable machine learning for biomarker identification and clinically interpretable pregnancy prediction	AI Supporting Evidence

Table 1 (continued)

Ref	Author (Reference)	Study Classification	Evidence Category	Primary Contribution to the Review	Quantitative AI Analysis
68	Wang et al. Wang [2025b]	Omics-based Artificial Intelligence	Category I – Primary AI Prediction Study	Omics-driven artificial intelligence models for early prediction of pregnancy complications	AI Performance Synthesis
69	He et al. He [2025]	Mechanistic Biology	Category IV – Mechanistic & Biomarker Study	Role of mitophagy and mitochondrial dysfunction in pregnancy complications	Mechanistic Evidence Synthesis
70	Qiao et al. Qiao et al. [2025]	Machine Learning	Category I – Primary AI Prediction Study	Inflammation-based machine learning model for first-trimester prediction of preeclampsia	AI Performance Synthesis
71	Liang et al. Liang et al. [2025b]	Machine Learning	Category I – Primary AI Prediction Study	Comprehensive first-trimester machine learning model for prediction of preeclampsia	AI Performance Synthesis
72	Ma et al. Ma et al. [2025]	Machine Learning	Category I – Primary AI Prediction Study	Machine learning pipeline for improving prediction of preeclampsia	AI Performance Synthesis
73	Zhu et al. Zhu et al. [2026]	Machine Learning	Category I – Primary AI Prediction Study	Integrative machine learning model for first-trimester prediction of preeclampsia	AI Performance Synthesis
74	Kim et al. Kim et al. [2026]	Machine Learning Methodology	Category II – AI Supporting & Translational Study	Machine learning strategies for handling missing clinical data in preeclampsia prediction	AI Supporting Evidence
75	Lei et al. Lei et al. [2026]	Genomic Artificial Intelligence	Category I – Primary AI Prediction Study	DNA methylation-based machine learning model for prediction of pregnancy complications	AI Performance Synthesis
76	Luo et al. Luo et al. [2026]	Clinical Outcomes	Category III – Clinical & Epidemiological Study	Maternal outcomes associated with gestational diabetes mellitus	Clinical Evidence Synthesis
77	Wang et al. Wang and Chen [2026]	Clinical Outcomes	Category III – Clinical & Epidemiological Study	Maternal and neonatal outcomes associated with gestational diabetes mellitus	Clinical Evidence Synthesis
78	Wu et al. Wu and Chen [2026]	Clinical Epidemiology	Category III – Clinical & Epidemiological Study	Risk factors for preterm birth in pregnancies complicated by gestational diabetes mellitus	Clinical Evidence Synthesis

Table 1 (continued)

Ref	Author (Reference)	Study Classification	Evidence Category	Primary Contribution to the Review	Quantitative AI Analysis
79	Weir et al. Weir [2026]	Clinical Epidemiology	Category III – Clinical & Epidemiological Study	Combined effects of prepregnancy obesity and gestational diabetes on pregnancy outcomes	Clinical Evidence Synthesis
80	Mensah et al. Mensah [2026]	Clinical Epidemiology	Category III – Clinical & Epidemiological Study	Association of body mass index and obstructive sleep apnea with risk of preeclampsia	Clinical Evidence Synthesis
81	Tagami et al. Tagami [2026]	Clinical Epidemiology	Category III – Clinical & Epidemiological Study	Advanced maternal age as a clinical risk factor for hypertensive disorders of pregnancy	Clinical Evidence Synthesis
82	Hughes et al. Hughes [2026]	Clinical Epidemiology	Category III – Clinical & Epidemiological Study	Population trends and maternal age distribution in hypertensive disorders of pregnancy	Clinical Evidence Synthesis
83	Rezaeinejad et al. Rezaeinejad [2026]	Systematic Review	Category III – Clinical & Epidemiological Study	Association between maternal smoking and the risk of preeclampsia	Clinical Evidence Synthesis
84	Iwama et al. Iwama [2026]	Clinical Cohort Study	Category III – Clinical & Epidemiological Study	Association between maternal birth weight and pregnancy complications	Clinical Evidence Synthesis
85	Keitaanpaa et al. Keitaanpaa [2026]	Longitudinal Cohort Study	Category III – Clinical & Epidemiological Study	Long-term cardiovascular risk following preeclampsia	Clinical Evidence Synthesis
86	Yang et al. Yang [2026]	Clinical Review	Category III – Clinical & Epidemiological Study	Cardiovascular complications following pregnancy-related disorders	Clinical Evidence Synthesis
87	Qiao et al. Qiao [2026]	Clinical Review	Category III – Clinical & Epidemiological Study	Low-dose aspirin for prevention of preeclampsia in high-risk pregnancies	Clinical Evidence Synthesis
88	Safavi Ardabili et al. Safavi Ardabili [2026]	Clinical Review	Category III – Clinical & Epidemiological Study	Management strategies for gestational diabetes mellitus and associated pregnancy outcomes	Clinical Evidence Synthesis

Table 1 (continued)

Ref	Author (Reference)	Study Classification	Evidence Category	Primary Contribution to the Review	Quantitative AI Analysis
89	Schoenaker et al. Schoenaker [2026]	Population-Based Cohort Study	Category III – Clinical & Epidemiological Study	Impact of type 2 diabetes on pregnancy outcomes	Clinical Evidence Synthesis
90	Garcia et al. de Alba Graue [2026]	Systematic Review	Category III – Clinical & Epidemiological Study	Association between endometriosis and the risk of preeclampsia and adverse pregnancy outcomes	Clinical Evidence Synthesis
91	Ye et al. Ye [2025]	Population-Based Cohort Study	Category III – Clinical, Epidemiological & Outcome Study	Association between gestational diabetes mellitus and subsequent risk of preeclampsia	Clinical Evidence Synthesis
92	Li et al. Li [2025]	Population-Based Cohort Study	Category III – Clinical, Epidemiological & Outcome Study	Joint epidemiological analysis of gestational diabetes mellitus and preeclampsia	Clinical Evidence Synthesis
93	Wu et al. Wu et al. [2025c]	Twin Cohort Study	Category III – Clinical, Epidemiological & Outcome Study	Shared maternal and fetal risks associated with gestational diabetes mellitus and preeclampsia	Clinical Evidence Synthesis
94	Tang et al. Tang [2025]	Twin Cohort Study	Category III – Clinical, Epidemiological & Outcome Study	Pregnancy outcomes associated with coexisting gestational diabetes mellitus and preeclampsia	Clinical Evidence Synthesis
95	Umer et al. Umer [2025]	Retrospective Cohort Study	Category III – Clinical, Epidemiological & Outcome Study	Maternal and neonatal outcomes associated with gestational diabetes mellitus and hypertensive disorders of pregnancy	Clinical Evidence Synthesis
96	Syngelaki et al. Syngelaki et al. [2025]	Prospective Cohort Study	Category III – Clinical, Epidemiological & Outcome Study	Large-scale cohort evaluating maternal risk factors for gestational diabetes mellitus and preeclampsia	Clinical Evidence Synthesis
97	Abdi et al. Abdi [2025]	Clinical Epidemiology	Category III – Clinical, Epidemiological & Outcome Study	Maternal obesity as a shared risk factor for gestational diabetes mellitus and preeclampsia	Shared Risk Factor Synthesis
98	van Niekerk et al. van Niekerk et al. [2021]	Mechanistic Clinical Study	Category IV – Mechanistic & Biomarker Study	Insulin resistance as a shared metabolic mechanism linking gestational diabetes mellitus and preeclampsia	Mechanistic Evidence Synthesis

Table 1 (continued)

Ref	Author (Reference)	Study Classification	Evidence Category	Primary Contribution to the Review	Quantitative AI Analysis
99	Ray et al. Ray et al. [2024]	Mechanistic Review	Category IV – Mechanistic & Biomarker Study	Inflammatory pathways underlying gestational diabetes mellitus and preeclampsia	Mechanistic Evidence Synthesis
100	Petkova-Parlapanska et al. Petkova-Parlapanska [2025]	Mechanistic Review	Category IV – Mechanistic & Biomarker Study	Oxidative stress mechanisms contributing to gestational diabetes mellitus and preeclampsia	Mechanistic Evidence Synthesis
101	Furuta et al. Furuta [2026]	Mechanistic Biology	Category IV – Mechanistic & Biomarker Study	Autophagy dysregulation and placental mechanisms underlying gestational diabetes mellitus and preeclampsia	Mechanistic Evidence Synthesis
102	Tossetta et al. Tossetta [2026]	Placental Biology	Category IV – Mechanistic & Biomarker Study	Placental dysfunction and angiogenic imbalance associated with pregnancy complications	Mechanistic Evidence Synthesis
103	Fiskaa et al. Fiskaa [2026]	Placental Biology	Category IV – Mechanistic & Biomarker Study	Placental vascular pathology and mechanisms contributing to preeclampsia	Mechanistic Evidence Synthesis
104	Zhang et al. Zhang et al. [2025b]	Biomarker Study	Category IV – Mechanistic & Biomarker Study	Evaluation of angiogenic biomarkers (sFlt-1/PlGF) for prediction of pregnancy complications	Biomarker Evidence Synthesis
105	Qi et al. Qi et al. [2025]	Biomarker Study	Category IV – Mechanistic & Biomarker Study	Clinical significance of placental growth factor in preeclampsia	Biomarker Evidence Synthesis
106	Chen et al. Chen [2025b]	Transcriptomics	Category IV – Mechanistic & Biomarker Study	Placental transcriptomic signatures associated with gestational diabetes mellitus and preeclampsia	Mechanistic Evidence Synthesis
107	Zhang et al. Zhang [2025]	Lipidomics	Category IV – Mechanistic & Biomarker Study	Lipidomic biomarkers associated with gestational diabetes mellitus and preeclampsia	Mechanistic Evidence Synthesis
108	Sun et al. Sun et al. [2025]	Inflammatory Biomarkers	Category IV – Mechanistic & Biomarker Study	Inflammatory biomarkers associated with pregnancy complications	Mechanistic Evidence Synthesis

Table 1 (continued)

Ref	Author (Reference)	Study Classification	Evidence Category	Primary Contribution to the Review	Quantitative AI Analysis
109	Guo et al. Guo [2024]	Clinical Biomarkers	Category IV – Mechanistic & Biomarker Study	Triglyceride-glucose index as a metabolic biomarker for prediction of pregnancy complications	Biomarker Evidence Synthesis
110	Bigdeli et al. Bigdeli et al. [2025]	Clinical Biomarkers	Category IV – Mechanistic & Biomarker Study	Clinical biomarkers associated with gestational diabetes mellitus prediction	Biomarker Evidence Synthesis
111	Dmitrenko et al. Dmitrenko [2025]	Molecular Biology	Category IV – Mechanistic & Biomarker Study	Role of SIRT1 signalling pathways in gestational diabetes mellitus and preeclampsia	Mechanistic Evidence Synthesis
112	Szukiewicz et al. Szukiewicz [2026]	Molecular Biology	Category IV – Mechanistic & Biomarker Study	Galectin-mediated placental mechanisms in pregnancy complications	Mechanistic Evidence Synthesis
113	Lu et al. Zuo et al. [2025]	Microbiome	Category IV – Mechanistic & Biomarker Study	Gut microbiota alterations associated with gestational diabetes mellitus and preeclampsia	Mechanistic Evidence Synthesis
114	Zhou et al. Zhou et al. [2024a]	Single-cell Transcriptomics	Category IV – Mechanistic & Biomarker Study	Single-cell transcriptomic profiling of placental tissue in pregnancy complications	Mechanistic Evidence Synthesis
115	He et al. He [2025]	Cellular Biology	Category IV – Mechanistic & Biomarker Study	Mitophagy and mitochondrial dysfunction associated with pregnancy complications	Mechanistic Evidence Synthesis
116	Qiao et al. Qiao et al. [2025]	Inflammatory Biomarkers	Category IV – Mechanistic & Biomarker Study	Inflammatory pathways supporting biologically informed prediction models	Mechanistic Evidence Synthesis
117	Wang et al. Wang [2025b]	Multi-omics	Category IV – Mechanistic & Biomarker Study	Integration of multi-omics data supporting precision prediction of pregnancy complications	Mechanistic Evidence Synthesis
118	Kabir et al. Kabir et al. [2024]	Biomarker Evidence	Category IV – Mechanistic & Biomarker Study	Systematic evaluation of biomarkers associated with preeclampsia among women with gestational diabetes mellitus supporting biomarker-based prediction	Mechanistic Evidence Synthesis

Table 1 (continued)

Ref	Author (Reference)	Study Classification	Evidence Category	Primary Contribution to the Review	Quantitative AI Analysis
119	Li et al. Li [2025]	Disease Association	Category III – Clinical, Epidemiological & Outcome Study	Combined epidemiological evidence linking gestational diabetes mellitus and preeclampsia	Clinical Evidence Synthesis
120	Ye et al. Ye [2025]	Disease Association	Category III – Clinical, Epidemiological & Outcome Study	Population-based evidence supporting the shared occurrence of gestational diabetes mellitus and preeclampsia	Clinical Evidence Synthesis

Supplementary Table S2

This supplementary Table 2 presents the study-level PROBAST risk-of-bias assessment for the 47 primary AI prediction studies included in the quantitative evidence synthesis. Risk of bias was evaluated across the four PROBAST domains (Participants, Predictors, Outcome, and Analysis), together with the overall judgement.

Table 2: Study-level PROBAST assessment of the 47 primary AI prediction studies included in the quantitative evidence synthesis.

#	Study	Participants	Predictors	Outcome	Analysis	Overall
1	Guo et al. [2025]	Low	Low	Low	Unclear	Unclear
2	Du et al. [2026]	Low	Low	Low	Unclear	Unclear
3	Hassan et al. [2025]	Unclear	Low	Low	Unclear	Unclear
4	Cardenas-Urrelo et al. [2026]	Low	Low	Low	Unclear	Unclear
5	Sun et al. [2022]	Low	Low	Low	Low	Low
6	Sun et al. [2023]	Low	Low	Low	Unclear	Unclear
7	Arora et al. [2025]	Low	Low	Low	Low	Low
8	Li et al. [2026]	Low	Low	Low	Low	Low
9	Dong et al. [2026]	Low	Low	Low	Unclear	Unclear
10	Liao et al. [2022]	Unclear	Low	Low	Low	Unclear
11	Butler et al. [2024a]	Low	Low	Low	Low	Low
12	Wang et al. [2024]	Low	Low	Low	Unclear	Unclear
13	Zhou et al. [2024a]	Low	Low	Low	Low	Low
14	Eberhard et al. [2024]	Low	Low	Low	Low	Low
15	Narayanan et al. [2026]	Low	Low	Low	Low	Low
16	Wu et al. [2025a]	Low	Low	Low	Low	Low
17	Zheng [2022]	Low	Low	Low	Low	Low

Table 2 (continued)

#	Study	Participants	Predictors	Outcome	Analysis	Overall
18	Zhou et al. [2024b]	Low	Low	Low	Low	Low
19	Kurt et al. [2023]	Low	Low	Low	Low	Low
20	Bennett et al. [2022]	Unclear	Low	Low	Unclear	Unclear
21	Eberhard et al. [2025]	Low	Low	Low	Low	Low
22	Liang et al. [2025a]	Unclear	Low	Low	High	High
23	Segal [2020]	Low	Low	Low	Unclear	Unclear
24	Winn [2020]	Low	Low	Low	Unclear	Unclear
25	Zhang et al. [2025a]	Low	Low	Low	Unclear	Unclear
26	Wang [2025a]	High	Low	Low	High	High
27	Zhang [2022]	Unclear	Low	Low	Unclear	Unclear
28	Pinto et al. [2023]	Low	Low	Low	Unclear	Unclear
29	Wang [2022]	Low	Low	Low	Unclear	Unclear
30	Xiao [2020]	Low	Low	Low	Unclear	Unclear
31	Yang [2020]	Low	Low	Low	Unclear	Unclear
32	Guo et al. [2020]	Low	Low	Low	Unclear	Unclear
33	Teede [2023]	Unclear	Low	Low	Unclear	Unclear
34	Li [2020]	Low	Low	Low	Unclear	Unclear
35	Hu [2021]	Low	Low	Low	Unclear	Unclear
36	Wang [2023]	Low	Low	Low	Unclear	Unclear
37	Syngelaki et al. [2025]	Low	Low	Low	Unclear	Unclear
38	Lu et al. [2024]	High	Low	Low	High	High
39	Deng et al. [2024]	Unclear	Low	Low	Unclear	Unclear
40	Zhang [2026]	Low	Low	Low	Low	Low
41	Metz [2021]	Unclear	Low	Low	Unclear	Unclear
42	Nicolaides [2022]	Unclear	Low	Low	Unclear	Unclear
43	Mustafa et al. [2025]	Low	Low	Low	Unclear	Unclear
44	Welsh [2022]	High	Low	Low	High	High
45	Kaya et al. [2024a]	Unclear	Low	Low	Unclear	Unclear
46	Aminifar [2022]	Low	Low	Low	Unclear	Unclear
47	Leung [2023]	Unclear	Low	Low	Low	Unclear

References

Juan Guo, Yuhong Huang, Zhiwei Zhang, Baoqiang Shi, Shuxian Xi, Yuanyuan Mai, Yan Liang, Zhizhen Guo, and Lantian Shang. Development and validation of a multi-modality system combining radiomics and deep learning for predicting mid-pregnancy complications and enabling timely pregnancy care. *Frontiers in Pediatrics*, 13:1716073, 2025. ISSN 2296-2360. doi: 10.3389/fped.2025.1716073. URL `https://doi.org/10.3389/fped.2025.1716073`.

YR Du, C Ji, J Jiao, F Xin, YY Ren, ZW Xia, Y Guo, and JQ Zhou. Multimodal deep learning fusion model for assessment of fetal lung development in gestational diabetes mellitus and pre-eclampsia. *Journal Placeholder*, 2026. doi: 10.3389/fendo.2026.1832468. 2.

Ahmad Hassan, Saima Gulzar Ahmad, Tassawar Iqbal, Ehsan Ullah Munir, Kashif Ayyub, and Naeem Ramzan. Enhanced model for gestational diabetes mellitus prediction using a fusion technique of multiple algorithms with explainability. *International Journal of Computational Intelligence Systems*, 18(1):47, 2025. doi: 10.1007/s44196-025-00760-4. 7.

Rosa Cardenas-Urrelo, Dino Quinteros-Navarro, Carmina Tang, Ynés Torres-Flores, Yolanda Navarro, Melissa Soto, Ana Maguiña, and Marina Huamantumba. Early prediction of preeclampsia using a multimodal machine learning model integrating clinical, hemodynamic, and biochemical data. *Ingénierie des Systèmes d'Information*, 31(3):985–999, 2026. doi: 10.18280/isi.310328.

Hongshuang Sun, Jing Jiao, Yunyun Ren, Yi Guo, and Yuanyuan Wang. Model application to quantitatively evaluate placental features from ultrasound images with gestational diabetes. *Journal of Clinical Ultrasound*, 50(9):1284–1292, 2022. doi: 10.1002/jcu.23233. 18.

Hongshuang Sun, Jing Jiao, Yunyun Ren, Yi Guo, and Yuanyuan Wang. Multimodal fusion model for classifying placenta ultrasound imaging in pregnancies with hypertension disorders. *Pregnancy Hypertension*, 31:31–38, 2023. doi: 10.1016/j.preghy.2022.12.003.

Urvashi Arora, Palani Vigneshwar, Munagala Krishna Sai, Reena Yadav, Debarka Sengupta, and Manisha Kumar. Ultrasound placental image texture analysis using artificial intelligence and deep learning models to predict hypertension in pregnancy. *International Journal of Gynecology & Obstetrics*, 171(3):1294–1301, 2025. doi: 10.1002/ijgo.70306.

Wei Li, Tao Xu, Yang He, Haotian Yuan, Wenyu Du, Wei Feng, and Jianqi Zhang. Value of multimodal ultrasound assessment of placental dysfunction in gestational diabetes mellitus: A prospective study. *Quantitative Imaging in Medicine and Surgery*, 2026. doi: 10.21037/qims-2025-aw-2103. 11.

Xuran Dong, Jinyue Dai, Zhuxin Xiong, Tianchen Wu, Yuan Wei, and Xiaofei Wang. A multimodal deep learning model for early risk prediction of preeclampsia integrating non-invasive fundus photography and longitudinal time-series clinical data. In *Investigative Ophthalmology & Visual Science*, volume 67, page 2309, 2026. doi: 10.1167/iovs.67.7.2309. URL `https://iovs.arvojournals.org/article.aspx?articleid=2813720`. ARVO Annual Meeting Abstract.

Yihong Liao, Tiantian Sun, Lei Jiang, Zhiyong Zhao, Tiantian Liu, Zhaoxin Qian, Yan Sun, Yan Zhang, and Di Wu. Detecting abnormal placental microvascular flow in maternal and fetal diseases based on flow-compensated and non-compensated intravoxel incoherent motion imaging. *Placenta*, 122:44–52, 2022. doi: 10.1016/j.placenta.2022.01.010. 17.

Liam Butler, Fatma Gunturkun, Lokesh Chinthala, Ibrahim Karabayir, Mohammad S. Tootooni, Berna Bakir-Batu, Turgay Celik, Oguz Akbilgic, and Robert L. Davis. Ai-based preeclampsia detection and prediction with electrocardiogram data. *Frontiers in Cardiovascular Medicine*, 11:1360238, 2024a. doi: 10.3389/fcvm.2024.1360238.

Yipeng Wang, Pei Sun, Zicheng Zhao, Yousheng Yan, Wentao Yue, Kai Yang, Ruixia Liu, Hui Huang, Yinan Wang, Yin Chen, Nan Li, Hailong Feng, Jing Li, Yifan Liu, Yujiao Chen, Bairong Shen, Lijian Zhao, and Chenghong Yin. Identify gestational diabetes mellitus by deep learning model from cell-free dna at the early gestation stage. *Briefings in Bioinformatics*, 25(1):bbad492, 2024. doi: 10.1093/bib/bbad492.

Huien Zhou, Wanming Chen, Chen Chen, Yanying Zeng, Jialin Chen, Jianru Lin, Kun He, and Xinmin Guo. Predictive value of ultrasonic artificial intelligence in placental characteristics of early pregnancy for gestational diabetes mellitus. *Frontiers in Endocrinology*, 15:1344666, 2024a. doi: 10.3389/fendo.2024.1344666.

Benjamin W. Eberhard, Kathryn J. Gray, David W. Bates, and Vesela P. Kovacheva. Deep survival analysis for interpretable time-varying prediction of preeclampsia risk. *Journal of Biomedical Informatics*, 154:104688, 2024. doi: 10.1016/j.jbi.2024.104688. 16.

Anju Narayanan, Praveen Sankaran, Uma V. Sankar, Simi Kurian, and Teslin John. Explainable tabnet for gestational diabetes prediction with physician-in-the-loop and multi-site clinical validation. *International Journal of Medical Informatics*, 195:106488, 2026. doi: 10.1016/j.ijmedinf.2026.106488. 37.

Yuxuan Wu, Lixia Shen, Lanqin Zhao, Xiaohong Lin, Miaohong Xu, Zhenjun Tu, Yihong Huang, Lingyi Kong, Zhenzhe Lin, Duoru Lin, Lixue Liu, Xun Wang, Zizheng Cao, Xi Chen, Shengmei Zhou, Weiling Hu, Yunjian Huang, Shiyuan Chen, Meimei Dongye, Xulin Zhang, Dongni Wang, Danli Shi, Zilian Wang, Xiaohang Wu, and Haotian Lin. Noninvasive early prediction of preeclampsia in pregnancy using retinal vascular features. *NPJ Digital Medicine*, 8(1):188, 2025a. doi: 10.1038/s41746-025-01582-6. 43.

D. Zheng. Comparison of machine learning and logistic regression models for predicting preeclampsia. *Frontiers in Medicine*, 2022.

Tian Zhou, Shijie Gu, Fei Shao, Peng Li, Yang Wu, Jun Xiong, Bo Wang, Cheng Zhou, Peng Gao, and Xun Hua. Prediction of preeclampsia from retinal fundus images via deep learning in singleton pregnancies: A prospective cohort study. *Journal of Hypertension*, 42(8):1630–1638, 2024b. doi: 10.1097/HJH.0000000000003658. 60.

Bilal Kurt, Burak Gürlek, Selim Keskin, Selçuk Özdemir, Özlem Karadeniz, İlknur Buçan Kırkbir, Tuğba Kurt, Serkan Ünsal, Ceyda Kart, Nilgün Baki, and Koray Turhan. Prediction of gestational diabetes using deep learning and bayesian optimization and traditional machine learning techniques. *Medical & Biological Engineering & Computing*, 61(11):2963–2977, 2023. doi: 10.1007/s11517-023-02800-7. 62.

Rebecca Bennett, Zuber D. Mulla, Pranav Parikh, Andrea Hauspurg, and Talayeh Razzaghi. An imbalance-aware deep neural network for early prediction of preeclampsia. *PLOS ONE*, 17(4):e0266042, 2022. doi: 10.1371/journal.pone.0266042. 68.

Benjamin W. Eberhard, Rachel Y. Cohen, Nicole Wheeler, Rachel Kleinlein, Julia Rigoni, David W. Bates, Kathryn J. Gray, and Vesela P. Kovacheva. Development and validation of an interpretable longitudinal preeclampsia risk prediction using machine learning. *PLOS ONE*, 20(5):e0323873, 2025. doi: 10.1371/journal.pone.0323873. 86.

Hong Liang, Xia Zhao, Yan Zhang, Yan Wu, Hao Wu, Zhen Zhang, and Yan He. A comprehensive first-trimester predictive model for preeclampsia based on multi-indicators and machine learning: A retrospective single-center study. *Medicine*, 104 (15):e45555, 2025a. doi: 10.1097/MD.0000000000045555. 89.

Nitzan Shalom Artzi; Smadar Shilo; Eran Hadar; Hagai Rossman; Shiri Barbash-Hazan; Avi Ben-Haroush; Ran D. Balicer; Becca Feldman; Arnon Wiznitzer; Eran Segal. Prediction of gestational diabetes based on nationwide electronic health records. *Nature Medicine*, 2020. doi: 10.1038/s41591-019-0724-8. AI001.

Ivana Maric; Abraham Tsur; Nima Aghaeepour; Andrea Montanari; David K. Stevenson; Gary M. Shaw; Virginia D. Winn. Early prediction of preeclampsia via machine learning. *AJOG MFM*, 2020. doi: 10.1016/j.ajogmf.2020.100100. AI002.

Jiani Zhang, Qi Cao, Chihui Mao, Jinfeng Xu, Yaqian Li, Yi Mu, Guiqiong Huang, Daijuan Chen, Xixi Deng, Tingting Xu, Fan Zhou, and Xiaodong Wang. Development and validation of a prediction model for gestational diabetes mellitus risk among women from 8 to 14 weeks of gestation in western china. *BMC Pregnancy and Childbirth*, 25:385, 2025a. doi: 10.1186/s12884-025-07442-1. AI003.

Qun Ji; Lan Gao; Haiwei Liu; Xiaofang Chen; Boxia Fu; Yingbei Lin; Fei Wang. Early prediction of gestational diabetes mellitus using machine learning-integrated metabolomic and clinical features. *Frontiers in Endocrinology*, 2025a. AI004.

Tao Zheng; Weiping Ye; Xipeng Wang; Xiaoyong Li; Jun Zhang; Julian Little; Lixia Zhou; Lin Zhang. A simple model to predict risk of gestational diabetes mellitus from 8 to 20 weeks of gestation in chinese women. *Scientific Reports*, 2022. doi: 10.1186/s12884-019-2374-8. AI008.

Yishay Pinto, Sigal Frishman, Sondra Turjeman, Adi Eshel, Meital Nuriel-Ohayon, Oshrit Shrossel, Oren Ziv, William Walters, Julie Parsonnet, Catherine Ley, Elizabeth L. Johnson, Krithika Kumar, Ron Schweitzer, Soliman Khatib, Faiga Magzal, Efrat Muller, Snait Tamir, Kinneret Tenenbaum-Gavish, Samuli Rautava, Seppo Salminen, Erika Isolauri, Or Yariv, Yoav Peled, Eran Poran, Joseph Pardo, Rony Chen, Moshe Hod, Elhanan Borenstein, Ruth E. Ley, Betty Schwartz, Yoram Louzoun, Eran Hadar, and Omry Koren. Gestational diabetes is driven by microbiota-induced inflammation months before diagnosis. *Gut*, 72(5):918–928, 2023. doi: 10.1136/gutjnl-2022-328406.

Yanran Du; Zhou Fang; Jing Jiao; Guannan Xi; Chen Zhu; Yunyun Ren; Yi Guo; Yuanyuan Wang. Application of ultrasound-based radiomics technology in fetal lung texture analysis in pregnancies complicated by gestational diabetes or preeclampsia. *Ultrasound in Obstetrics & Gynecology*, 2022. doi: 10.1002/uog.22037. AI026.

Yunzhen Ye; Yu Xiong; Qiongjie Zhou; Jiangnan Wu; Xiaotian Li; Xirong Xiao. Comparison of machine learning methods and conventional logistic regressions for predicting gestational diabetes using routine clinical data: A retrospective cohort study. *Journal of Diabetes Research*, 2020. AI009.

M. Ranjbar et al. Machine learning models for predicting preeclampsia: A systematic review. *BMC Pregnancy and Childbirth*, 24:22, 2024. doi: 10.1186/s12884-023-06220-1.

S. Ahmed et al. Artificial intelligence for early detection of preeclampsia and gestational diabetes mellitus: A systematic review of diagnostic performance. *Cureus*, 17 (8):e92585, 2025. doi: 10.7759/cureus.92585.

Lu Liu, Qixuan Zhu, Yichi Zong, Xueyuan Chen, Wei Zhang, and Jun Wang. Machine learning prediction models for preeclampsia: Systematic review and meta-analysis. *Journal of Medical Internet Research*, 28:e78714, 2026. doi: 10.2196/78714.

S. S. Aljameel, F. A. Alzahrani, A. Alshamrani, et al. Prediction of preeclampsia using machine learning and deep learning models: A review. *Big Data and Cognitive Computing*, 7(1):32, 2023. doi: 10.3390/bdcc7010032.

T. Özcan and M. Peker. Preeclampsia prediction via machine learning: A systematic literature review. *The Journal of Maternal-Fetal & Neonatal Medicine*, 2025. doi: 10.1080/20476965.2024.2435845.

E. Kokori, G. Olatunji, N. Aderinto, and I. Muogbo. The role of machine learning algorithms in detection of gestational diabetes: A narrative review of current evidence. *Clinical Diabetes and Endocrinology*, 2024. doi: 10.1186/s40842-024-00176-7.

P. Popova. Artificial intelligence-based clinical decision support system for gestational diabetes management. *Frontiers in Endocrinology*, 2023.

Shivshanker Singh Patel. Explainable machine learning models to analyse maternal health. *Data & Knowledge Engineering*, 146:102198, 2023. doi: 10.1016/j.datak.2023.102198.

J. Chen. Explainable machine learning approaches for identifying key biomarkers in preeclampsia prediction. *Frontiers in Medicine*, 2025a.

Hannah D. Scott; Marrissa Buchan; Caylin Chadwick; Catherine J. Field; Nicole Letourneau; Tony Montina; Brenda M. Y. Leung; Gerlinde A. S. Metz. Metabolic dysfunction in pregnancy: Fingerprinting the maternal metabolome using proton nuclear magnetic resonance spectroscopy. *Endocrinology, Diabetes & Metabolism*, 2021. doi: 10.1002/edm2.201. AI024.

Guixue Hou; Ya Gao; Liona C. Poon; Yan Ren; Chunwei Zeng; Bo Wen; Argyro Syngelaki; Liang Lin; Jin Zi; Fengxia Su; Weiwei Xie; Fang Chen; Kypros H. Nicolaides. Maternal plasma diacylglycerols and triacylglycerols in the prediction of gestational diabetes mellitus. *BJOG*, 2022. doi: 10.1111/1471-0528.17297. AI025.

H. J. Mustafa, A. Khalil, A. Sotiriadis, et al. Prediction of hypertension and diabetes in twin pregnancy using machine learning model based on characteristics at first prenatal visit: National registry study. *Ultrasound in Obstetrics & Gynecology*, 2025. doi: 10.1002/uog.26534.

Daria Di Filippo; Marrwah Ahmadzai; Melissa Han Yiin Chang; Ksana Horgan; Ru Min Ong; Justine Darling; Mahmood Akhtar; Amanda Henry; Alec Welsh. Continuous glucose monitoring for the diagnosis of gestational diabetes mellitus: A pilot study. *Journal of Diabetes Research*, 2022. doi: 10.1155/2022/5142918. AI027.

Yeliz Kaya, Zafer Bütün, Çelik Özer, Ece Akça Salik, Tuğba Tahta, and Arzu Altun Yavuz. The early prediction of gestational diabetes mellitus by machine learning models. *BMC Pregnancy and Childbirth*, 24:547, 2024a. doi: 10.1186/s12884-024-06783-7. AI028.

Rasool Jader; Sadegh Aminifar. Predictive model for diagnosis of gestational diabetes in the kurdistan region by a combination of clustering and classification algorithms: An ensemble approach. *Applied Computational Intelligence and Soft Computing*, 2022. doi: 10.1155/2022/9749579. AI029.

Yun-Nam Chan; Pengpeng Wang; Ka-Him Chun; Judy Tsz-Shan Lum; Hang Wang; Yunhui Zhang; Kelvin Sze-Yin Leung. A machine learning approach for early prediction of gestational diabetes mellitus using elemental contents in fingernails. *Scientific Reports*, 2023. doi: 10.1038/s41598-023-31270-y. AI030.

H. Zhang. Development and validation of an integrated machine learning model for gestational diabetes prediction. *Frontiers in Endocrinology*, 2023.

Z. Sharifi-Heris. Machine learning-based prediction of pregnancy outcomes using wearable sensor data. *Frontiers in Digital Health*, 2023.

X. Liang. Continuous glucose monitoring-based machine learning models for predicting gestational diabetes outcomes. *The Lancet Regional Health – Western Pacific*, 2023.

K.H. Huang. Artificial neural network-based prediction of preeclampsia using clinical parameters. *Frontiers in Medicine*, 2022.

T. Li, M. Xu, Y. Wang, H. Tang, and H. Duan. Prediction model of preeclampsia using machine learning methods: a population-based cohort study. *Frontiers in Medicine*, 2024.

H.K. Ballard, X. Yang, A.D. Mahadevan, and D.J. Lemas. Five-feature machine learning models for predicting preeclampsia onset time using electronic health record data. *Journal of Medical Internet Research*, 2024.

S.A. Tiruneh, D.L. Rolnik, H.J. Teede, and J. Enticott. Comparative analysis of machine learning algorithms for prediction of preeclampsia risk. *International Journal of Medical Informatics*, 2024.

Y. Yu, W. Xu, S. Zhang, and S. Feng. Cell-free dna-based machine learning model for early prediction of preeclampsia. *Frontiers in Medicine*, 2024.

V.P. Kovacheva, B.W. Eberhard, R.Y. Cohen, and M. Maher. Preeclampsia prediction using machine learning and polygenic risk scores from clinical and genetic risk factors. *Hypertension*, 2024.

L. Butler, F. Gunturkun, and L. Chinthala. Ai-based preeclampsia detection and prediction using electrocardiogram data. *Frontiers in Medicine*, 2024b.

Y. Kaya, Z. Bütün, and Ö. Çelik. Risk assessment for preeclampsia in the preconception period based on maternal clinical history via machine learning methods. *Journal of Clinical Medicine*, 2024b.

Yuxuan Wu, Lixia Shen, Lanqin Zhao, Xiaohong Lin, Miaohong Xu, Zhenjun Tu, Yihong Huang, Lingyi Kong, Zhenzhe Lin, Duoru Lin, Lixue Liu, Xun Wang, Zizheng Cao, Xi Chen, Shengmei Zhou, Weiling Hu, Yunjian Huang, Shiyuan Chen, Meimei Dongye, Xulin Zhang, Dongni Wang, Danli Shi, Zilian Wang, Xiaohang Wu, Dongyu Wang, and Haotian Lin. Noninvasive early prediction of preeclampsia in pregnancy using retinal vascular features. *NPJ Digital Medicine*, 8:109, 2025b. doi: 10.1038/s41746-025-01582-6.

M. Adil, T.R. Kolarova, A.L. Doebley, and L.A. Chen. Preeclampsia risk prediction from prenatal cell-free dna screening. *Nature Medicine*, 2025.

Hesham Zaky, Eleni Fthenou, Luma Srour, Thomas Farrell, Mohammed Bashir, Nady El Hajj, and Tanvir Alam. Machine learning based model for the early detection of gestational diabetes mellitus. *BMC Medical Informatics and Decision Making*, 25:130, 2025. ISSN 1472-6947. doi: 10.1186/s12911-025-02947-3. URL `https://doi.org/10.1186/s12911-025-02947-3`. AI.

Q. Wang. Omics-driven machine learning models for early prediction of pregnancy-related disorders. *Frontiers in Immunology*, 2025b.

L. He. Mitophagy pathways in gestational diabetes mellitus and preeclampsia: molecular mechanisms and clinical implications. *European Journal of Medical Research*, 2025.

L. Qiao, Y. Zhang, and J. Wang. Machine learning-based prediction of preeclampsia using first-trimester inflammatory markers and red blood cell indices. *BMC Pregnancy and Childbirth*, 2025.

H. Liang, X. Zhao, and Y. Zhang. A comprehensive first-trimester predictive model for preeclampsia based on multi-indicators and machine learning. *Medicine*, 2025b.

Y. Ma, H. Lv, and X. Wang. Advancing preeclampsia prediction using a machine learning pipeline with resampling and ensemble models for imbalanced data. *BioData Mining*, 2025.

Y. Zhu, Y. Zhang, and C. Huang. First-trimester preeclampsia prediction model via integrative machine learning of maternal risk profiles and laboratory markers. *Genes & Diseases*, 2026.

J.W. Kim, N. Kim, J.Y. Kim, and H.J. Han. Handling missing data in machine learning-based prediction of preeclampsia: a robust framework. *Scientific Reports*, 2026.

J. Lei, H. Wu, H. Liu, and L.C. Poon. Dna methylation-based machine learning models for prediction of pregnancy-related hypertension disorders. *Hypertension*, 2026.

R. Luo, D.B. Fell, and D.J. Corsi. Risk of adverse maternal outcomes among pregnancies with gestational diabetes mellitus. *BMC Pregnancy and Childbirth*, 26:148, 2026.

C.W. Wang and H.H. Chen. Effect of gestational diabetes mellitus on maternal and neonatal outcomes: a population-based study. *Taiwanese Journal of Obstetrics and Gynecology*, 2026.

Y. Wu and L. Chen. Risk factors of preterm birth among pregnancies complicated by gestational diabetes mellitus. *Frontiers in Pediatrics*, 2026.

T.L. Weir. Impact of prepregnancy obesity and gestational diabetes on maternal and neonatal outcomes. *Diabetic Medicine*, 2026.

N.A. Mensah. Association of body mass index and obstructive sleep apnea with risk of preeclampsia. *American Journal of Perinatology*, 2026.

K. Tagami. Advanced maternal age and risk of hypertensive disorders of pregnancy: a cohort study. *Hypertension in Pregnancy*, 2026.

Z.H. Hughes. Maternal age distribution and trends in hypertensive disorders of pregnancy. *Circulation: Population Health and Outcomes*, 2026.

M. Rezaeinejad. Maternal smoking and risk of preeclampsia: a systematic analysis. *Pregnancy Hypertension*, 2026.

N. Iwama. Association between maternal birth weight and risk of pregnancy complications. *Journal of Obstetrics and Gynaecology Research*, 2026.

N. Keitaanpaa. Duration of preeclampsia and subsequent cardiovascular disease risk in women. *Hypertension*, 2026.

Y. Yang. Cardiovascular complications associated with pregnancy and long-term maternal health. *Journal of Clinical Investigation*, 2026.

Y. Qiao. Low-dose aspirin for prevention of preeclampsia in high-risk pregnancies: a randomized trial. *BMC Pregnancy and Childbirth*, 2026.

N. Safavi Ardabili. Management strategies for gestational diabetes mellitus and associated pregnancy outcomes. *Journal of Diabetes and Metabolic Disorders*, 2026.

D. Schoenaker. Impact of type 2 diabetes on pregnancy outcomes: a population-based analysis. *Diabetic Medicine*, 2026.

P.G. de Alba Graue. Association between endometriosis and risk of preeclampsia and adverse pregnancy outcomes. *Journal of Minimally Invasive Gynecology*, 2026.

Y. Ye. Risk factors for preeclampsia in pregnancies complicated by gestational diabetes mellitus. *British Journal of Hospital Medicine*, 2025.

H. Li. Combined effect of gestational diabetes mellitus and preeclampsia on maternal and neonatal outcomes. *Clinical and Experimental Obstetrics and Gynecology*, 2025.

Hanglin Wu, Lin Zhang, Zhen Xie, Hongxia Cai, Jindi Zhang, and Liming Yu. The impact of gestational diabetes mellitus on the development of preeclampsia in twin pregnancies: A retrospective cohort study conducted at a tertiary hospital. *International Journal of Women's Health*, 17:93–102, 2025c. doi: 10.2147/IJWH.S495443.

W.Z. Tang. Pregnancy outcomes in twin gestations complicated by gestational diabetes and hypertensive disorders. *Acta Diabetologica*, 2025.

A. Umer. Maternal and neonatal outcomes in pregnancies complicated by gestational diabetes mellitus and preeclampsia. *Cureus*, 2025.

Argyro Syngelaki, Alan Wright, Cristina Gomez Fernandez, Rea Mitsigiorgi, and Kypros H. Nicolaides. First-trimester prediction of gestational diabetes mellitus based on maternal risk factors. *Ultrasound in Obstetrics & Gynecology*, 65(5):633–641, 2025. doi: 10.1002/uog.29270. AI012.

F. Abdi. Prepregnancy obesity and risk of adverse pregnancy outcomes: a systematic review and meta-analysis. *Scientific Reports*, 2025.

Gustav van Niekerk, Claudia Christowitz, and Anna-Mart Engelbrecht. Insulin-mediated immune dysfunction in the development of preeclampsia. *Journal of Molecular Medicine*, 99(7):889–897, 2021. doi: 10.1007/s00109-021-02068-5.

G. W. Ray, Q. Zeng, P. Kusi, H. Zhang, T. Shao, T. Yang, Y. Wei, M. Li, X. Che, and R. Guo. Genetic and inflammatory factors underlying gestational diabetes mellitus: A review. *Frontiers in Endocrinology*, 15:1399694, 2024. doi: 10.3389/fendo.2024.1399694.

K. Petkova-Parlapanska. Oxidative stress pathways in gestational diabetes mellitus and preeclampsia. *International Journal of Molecular Sciences*, 2025.

A. Furuta. Autophagic dysfunction in the pathogenesis of preeclampsia. *Biomolecules*, 2026.

G. Tossetta. Placental mechanisms underlying gestational diabetes mellitus: molecular insights. *International Journal of Molecular Sciences*, 2026.

B.S. Fiskaa. Placental senescence markers and their role in gestational diabetes mellitus. *Placenta*, 2026.

L. Zhang, W. Li, X. Chi, Q. Sun, Y. Li, W. Xing, and G. Ding. Predictive performance of sflt-1, plgf and the sflt-1/plgf ratio for preeclampsia: A systematic review and meta-analysis. *Journal of Gynecology Obstetrics and Human Reproduction*, 2025b. doi: 10.1016/j.jogoh.2025.102925.

Guijie Qi, Ling Yao, Zhiming Liu, Wanru Guo, Heng Liu, Jinghua Zhang, Yulian He, and Tiancong Jiang. Placental growth factor as a predictive marker of preeclampsia in twin pregnancy. *Journal of Perinatal Medicine*, 53(2):149–157, 2025. doi: 10.1515/jpm-2024-0184.

Z. Chen. Placental transcriptomic alterations in gestational diabetes mellitus and preeclampsia. *Scientific Reports*, 2025b.

C. Zhang. Lipidomic and transcriptomic profiling reveals molecular mechanisms in gestational diabetes and preeclampsia. *Scientific Reports*, 2025.

Y. Sun, C. Shen, J. Li, W. Kang, X. Li, and W. Fan. Inflammatory indices in first trimester as predictors of gestational diabetes mellitus and adverse pregnancy outcomes. *American Journal of Reproductive Immunology*, 93:e70070, 2025. doi: 10.1111/aji.70070.

Y. Guo. Triglyceride-glucose index as a predictor of gestational diabetes mellitus: a cohort study. *Lipids in Health and Disease*, 2024.

Somayeh Kianian Bigdeli, Marjan Ghazisaedi, Seyed Mohammad Ayyoubzadeh, Sedigheh Hantoushzadeh, and Marjan Ahmadi. Predicting gestational diabetes mellitus in the first trimester using machine learning algorithms: A cross-sectional study at a hospital fertility health center in iran. *BMC Medical Informatics and Decision Making*, 25(1):3, 2025. doi: 10.1186/s12911-024-02799-3.

O. Dmitrenko. Sirt1 signaling and its role in the development of preeclampsia. *International Journal of Molecular Sciences*, 2025.

D. Szukiewicz. Galectin signaling pathways in gestational diabetes mellitus and placental dysfunction. *International Journal of Molecular Sciences*, 2026.

Zhihua Zuo, Shanshui Zeng, Xinyi Ou, Lijun Du, and Yongcan Guo. Gut microbiota changes in patients with hypertensive disorders and gestational diabetes in early pregnancy: A prospective cohort study. *BMC Pregnancy and Childbirth*, 25:603, 2025. doi: 10.1186/s12884-025-07964-8.

Y. Kabir et al. Biomarkers of pre-eclampsia in pregnant women with gestational diabetes mellitus: A systematic review. *Cureus*, 16(2):e53828, 2024. doi: 10.7759/cureus.53828.

Hongwei Liu; Jing Li; Junhong Leng; Hui Wang; Jinnan Liu; Weiqin Li; Hongyan Liu; Shuo Wang; Jun Ma; Juliana C. N. Chan; Zhijie Yu; Gang Hu; Changping Li; Xilin Yang. Machine learning risk score for prediction of gestational diabetes in early pregnancy in tianjin, china. *Diabetes/Metabolism Research and Reviews*, 2020. doi: 10.1002/dmrr.3397. AI011.

Zhiwei Guo, Fang Yang, Jun Zhang, Zhigang Zhang, Kun Li, Qi Tian, Hongying Hou, Cailing Xu, Qianwen Lu, Zhonglu Ren, Xiaoxue Yang, Zenglu Lv, Ke Wang, Xinping Yang, Yingsong Wu, and Xuexi Yang. Whole-genome promoter profiling of plasma dna exhibits diagnostic value for placenta-origin pregnancy complications. *Advanced Science*, 7(7):1901819, 2020. doi: 10.1002/advs.201901819.

Yitayeh Belsti; Lisa Moran; Lan Du; Aya Mousa; Kushan De Silva; Joanne Enticott; Helena Teede. Comparison of machine learning and conventional logistic regression-based prediction models for gestational diabetes in an ethnically diverse population; the monash gdm machine learning model. *International Journal of Medical Informatics*, 2023. doi: 10.1016/j.ijmedinf.2023.105228. AI013.

Yan Xiong; Lu Lin; Yu Chen; Stephen Salerno; Yi Li; Xiaoxi Zeng; Huafeng Li. Prediction of gestational diabetes mellitus in the first 19 weeks of pregnancy using machine learning techniques. *Journal of Maternal-Fetal & Neonatal Medicine*, 2020. doi: 10.1080/14767058.2020.1786517. AI014.

Wenqian Lu; Mingjuan Luo; Xiangnan Fang; Rong Zhang; Shanshan Li; Mengyang Tang; Xiangtian Yu; Cheng Hu. Discovery of metabolic biomarkers for gestational diabetes mellitus in a chinese population. *BMC Pregnancy and Childbirth*, 2021. doi: 10.1186/s12884-021-04295-2. AI017.

Xiaoqi Hu; Xiaolin Hu; Ya Yu; Jia Wang. Prediction model for gestational diabetes mellitus using the xg boost machine learning algorithm. *Frontiers in Endocrinology*, 2023. doi: 10.3389/fendo.2023.1105062. AI023.

Q. Lu, Y. Li, D. Ye, X. Yu, W. Huang, S. Zang, G. Jiang, et al. Longitudinal metabolomics integrated with machine learning identifies novel biomarkers of gestational diabetes mellitus. *Free Radical Biology and Medicine*, 2024. doi: 10.1016/j.freeradbiomed.2023.10.014. Longitudinal nested case-control study.

Yun Deng, Shujun Yi, Wenya Liu, Liping Yang, Lingyan Zhu, Qiang Zhang, Hangbiao Jin, Rongyan Yang, Rouyi Wang, and Nai-jun Tang. Identification of primary organophosphate esters contributing to enhanced risk of gestational diabetes mellitus based on a case-control study. *Environmental Science & Technology*, 2024. doi: 10.1021/acs.est.4c04180. AI022.

Guixi Wang; Wenfeng Zhang. Development and validation of a model integrating clinical and metabolomic markers for gestational diabetes mellitus prediction. *Frontiers in Medicine*, 2026. doi: 10.3389/fendo.2025.1687146. AI005.
